# Deletion of the Murine Cytochrome P450 *Cyp2j* Locus by Fused BAC-Mediated Recombination Identifies a Role for *Cyp2j* in the Pulmonary Vascular Response to Hypoxia

**DOI:** 10.1371/journal.pgen.1003950

**Published:** 2013-11-21

**Authors:** Guo Ling Zhou, Arkadi Beloiartsev, Binglan Yu, David M. Baron, Weihua Zhou, Rasma Niedra, Naifang Lu, Laurel T. Tainsh, Warren M. Zapol, Brian Seed, Kenneth D. Bloch

**Affiliations:** 1 Center for Computational and Integrative Biology, Massachusetts General Hospital, Boston, Massachusetts, United States of America; 2 Department of Anesthesia, Critical Care, and Pain Medicine, Massachusetts General Hospital, Boston, Massachusetts, United States of America; 3 Cardiovascular Research Center, Massachusetts General Hospital, Boston, Massachusetts, United States of America; University of Chicago, United States of America

## Abstract

Epoxyeicosatrienoic acids (EETs) confer vasoactive and cardioprotective functions. Genetic analysis of the contributions of these short-lived mediators to pathophysiology has been confounded to date by the allelic expansion in rodents of the portion of the genome syntenic to human *CYP2J2*, a gene encoding one of the principle cytochrome P450 epoxygenases responsible for the formation of EETs in humans. Mice have eight potentially functional genes that could direct the synthesis of epoxygenases with properties similar to those of CYP2J2. As an initial step towards understanding the role of the murine *Cyp2j* locus, we have created mice bearing a 626-kb deletion spanning the entire region syntenic to *CYP2J2*, using a combination of homologous and site-directed recombination strategies. A mouse strain in which the locus deletion was complemented by transgenic delivery of BAC sequences encoding human CYP2J2 was also created. Systemic and pulmonary hemodynamic measurements did not differ in wild-type, null, and complemented mice at baseline. However, hypoxic pulmonary vasoconstriction (HPV) during left mainstem bronchus occlusion was impaired and associated with reduced systemic oxygenation in null mice, but not in null mice bearing the human transgene. Administration of an epoxygenase inhibitor to wild-type mice also impaired HPV. These findings demonstrate that *Cyp2j* gene products regulate the pulmonary vascular response to hypoxia.

## Author Summary

In mice and humans, the CYP2J class of cytochrome P450 epoxygenases metabolizes arachidonic acid (AA) to epoxyeicosatrienoic acids (EETs), short-lived mediators with effects on both the pulmonary and systemic vasculature. Genetic dissection of CYP2J function to date has been complicated by allelic expansion in the rodent genome. In this study, the mouse chromosomal locus syntenic to human *CYP2J2*, containing eight presumed genes and two pseudogenes, was deleted via generation of a recombinant template created by homologous and site-specific recombination steps that joined two precursor bacterial artificial chromosomes (BACs). The *Cyp2j* null mice were subsequently complemented by transgenic delivery of BAC sequences encoding human CYP2J2. Hypoxic pulmonary vasoconstriction (HPV) and systemic oxygenation during regional alveolar hypoxia were unexpectedly found to be impaired in null mice, but not in null mice bearing the transgenic human allele, suggesting that *Cyp2j* products contribute to the pulmonary vascular response to hypoxia.

## Introduction

Human cytochrome P450 2J2 (CYP2J2) is abundant in cardiovascular tissues and pulmonary endothelium [1] and metabolizes arachidonic acid (AA) to epoxyeicosatrienoic acids (EETs) and hydroxyeicosatetraenoic acids (HETEs), short-lived mediators that have potent vascular protective properties [2]–[4]. The mouse chromosomal locus syntenic to human *CYP2J2* contains 10 genes (8 presumed genes and 2 pseudogenes) spanning 626 kb on chromosome 4. Gene clusters in the mouse that are syntenic to a single human gene are not uncommon, but their study is rarely straightforward. Mutant mice with short gene deletions can be generated through the conventional gene targeting strategies [5], but the deleted region rarely exceeds ten kilobases in most applications of the existing technology. Bacterial artificial chromosomes (BACs), which can have lengths up to ∼250 kb, have been used for gene targeting [6]–[8], but even in these cases the length of the BAC creates a formal upper limit for the size of the deletion. Deletion of a large DNA region has been accomplished by sequential introduction of loxP sites followed by the expression of Cre recombinase in embryonic stem cells [9]–[11]. However, it is difficult to distinguish loxP sites integrated into the same autosome from those integrated into separate autosomes, and Cre-mediated recombination has relatively low efficiency when the distance between loxP sites is great [9]–[11]. Here we describe a method to join BACs using prokaryotic integrases to create a deletion replica in *E. coli* that is subsequently used to target the murine locus.

*CYP2J2* products elicit a variety of effects including fibrinolysis, vasodilation, and inhibition of inflammation [12]–[14]. However, a definitive identification of the contributions of *Cyp2j* genes in the cardiovascular system has remained challenging due to the expansion of the locus in mice. Murine *Cyp2j* isoforms may act as epoxygenases and hydroxylases to metabolize AA into EETs and HETEs [15]. It has been shown that the pulmonary vasoconstrictor response to alveolar hypoxia is ablated in mice deficient for cytosolic phospholipase A2α (cPLA2α), an enzyme that lies upstream of Cyp2j and is responsible for liberation of AA from esterified forms of phospholipids in the cell membrane [16]. There are four pathways downstream of cPLA2α mediating the metabolism of AA, including the cyclooxygenase (COX), lipoxygenase (LO), epoxygenase, and ω-hydroxylase pathways. It has been shown that inhibition of COX or 5-LO pathways does not impair hypoxic pulmonary vasoconstriction (HPV) [17], [18]. Previous studies have demonstrated that products of CYP epoxygenases and hydroxylases can produce pulmonary vasoconstriction and vasodilation, respectively. However, it is unknown which cytochrome P450 is the major contributor to the regulation of HPV - a mechanism unique to the pulmonary vasculature, that diverts blood flow away from poorly ventilated lung regions, thereby preserving oxygenation of systemic blood [4], [16], [19]. The pulmonary vasoconstrictor response to alveolar hypoxia is crucial for maintaining arterial oxygenation during acute respiratory failure and lung injury. Due to the diversity of potential metabolites and the challenges associated with their measurement, stemming from their propensity for rapid metabolism and multiple functionally-relevant isomeric forms, it has been challenging to precisely identify which gene family and which eicosanoids are the most relevant modulators of HPV.

In this study, we describe a strategy to engineer large DNA fragments in bacteria and mammalian cells. We performed large scale ablation and human allelic complementation of the *Cyp2j* locus in mice using *E. coli* genetic techniques and bacterial artificial chromosome technology. Phenotypic characterization of the resulting *Cyp2j*-null and complemented mice showed that disruption of mouse *Cyp2j* genes did not alter pulmonary and systemic hemodynamic parameters at baseline. However, the increase in left lung pulmonary vascular resistance induced by selective left lung hypoxia was impaired in *Cyp2j*-null mice, but not in complemented mice, demonstrating that the *Cyp2j* genes contribute to hypoxic pulmonary vasoconstriction.

## Results

### Fusion of BACs using TP901 integrase in *E. coli*

**Figure 1:**
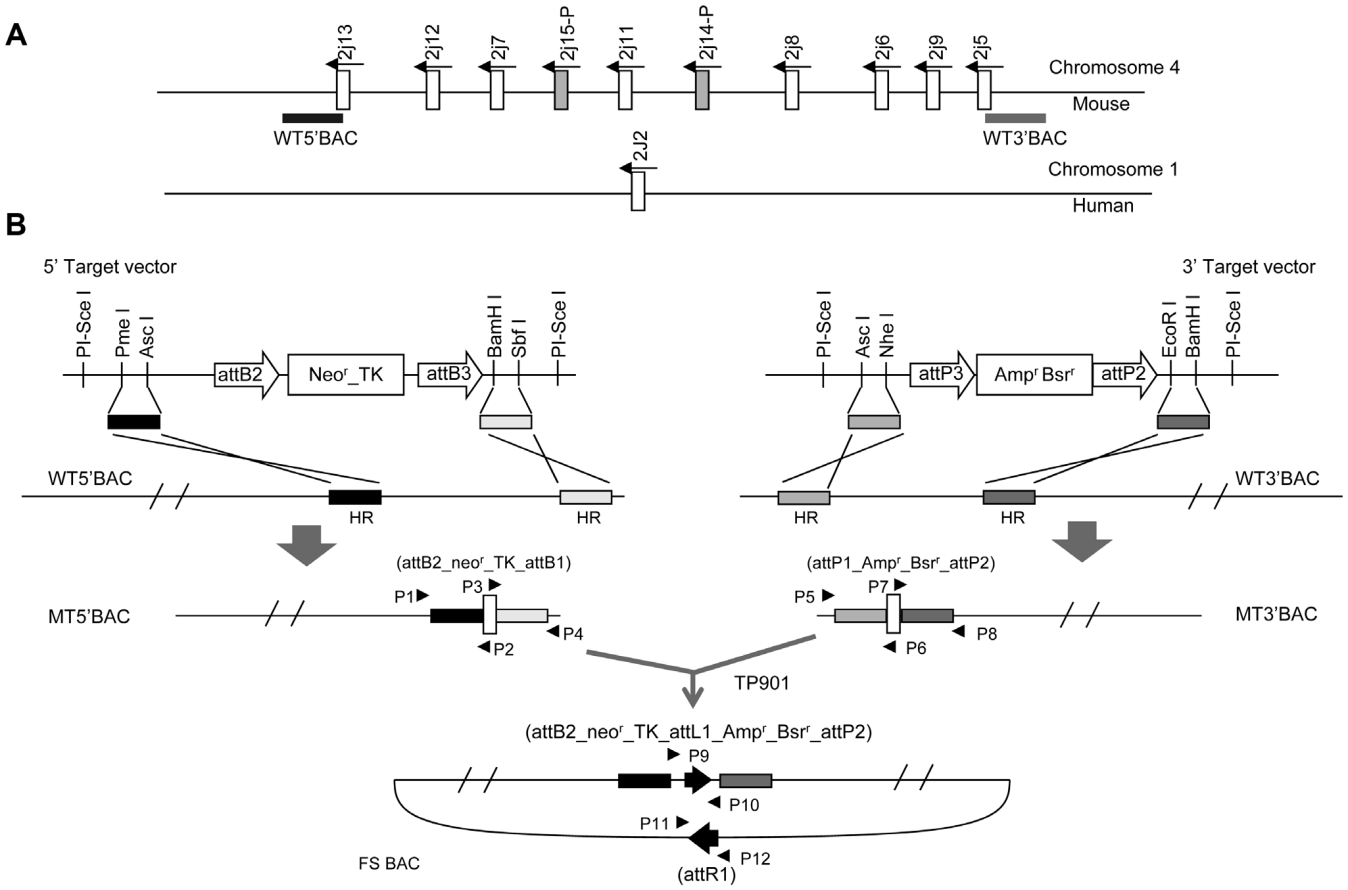
Construction of *Cyp2j* locus deletion replica. (A) Schematic map of mouse *Cyp2j* cluster and the human syntenic locus *CYP2J2*. (B) Construction strategy. The WT 5′BAC and WT 3′BAC (location shown on A) were modified using 5′ and 3′ targeting vectors, respectively, through homologous recombination in *E. coli*. The resultant MT5′BAC and MT3′BAC lack sequences between the recombination arms (HR) and have acquired selectable markers (neomycin resistance, thymidine kinase sensitivity, and blasticidin resistance) and integrase sites (attB1 and attP1 of TP901, attB2 and attP2 of R4). The fused BAC (FS BAC) was generated through site-specific recombination between attB3 and attP3 sites carried by the MT5′BAC and MT3′BAC, respectively, and mediated by TP901 integrase. Amp^r^, Ampicillin resistance; Bsr^r^, blasticidin resistance; TK, herpes simplex thymidine kinase; neo^r^, kanamycin/geneticin resistance.

Because no single BAC has been reported to span the entire mouse *Cyp2j* locus, two BACs that contain the termini of the *Cyp2j* gene cluster were separately modified to permit joining by site-specific recombination in *E. coli* (Figure 1A, 1B). Homologous arms were amplified from the BAC clones and subcloned into one targeting vector containing a kanamycin resistance element and the TP901-1 integrase *attB* site, and into another targeting vector containing an ampicillin resistance element and a TP901-1 *attP* site. Following homologous recombination, the selectable markers and integrase sites were integrated into the two BACs, forming MT5′BAC and MT3′BAC (Figure 1B), as identified using four PCR amplifications (using primers P1 to P8; Figure S1A). The PCR products were sequenced to confirm that the correct recombination products had formed.

The MT5′BAC and the MT3′BAC containing the termini of the mouse *Cyp2j* locus were then fused in *E. coli* by site-specific recombination. A plasmid expressing TP901 integrase under control of the *araBAD* promoter was introduced into a bacterial strain harboring the BAC containing the kanamycin resistance element and the TP901 *attB* site. TP901 expression was induced prior to creation of electrocompetent cells, and the modified BAC bearing ampicillin resistance was introduced. Following selection for resistance to both kanamycin and ampicillin the fused BAC resulting from integrase was identified by PCR. Two pairs of primers (P9 to P12 shown in Figure 1B) were used to identify the integration events (PCR results shown in Figure S1B). PCR products were sequenced to confirm the desired TP901 *attL* and *attR* sites had formed (representative sequences shown in Figure S1B). The correctly fused BAC (FS BAC) was digested with *Spe*I and *BamH*I to confirm that no unwanted rearrangements had occurred (Figure S1C).

### Excision of mouse *Cyp2j* gene cluster using a deletion replica in mouse ES cells

**Figure 2:**
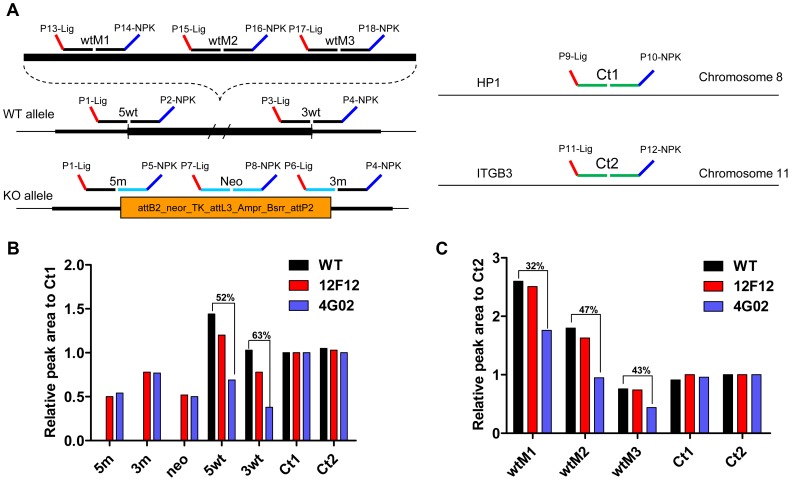
Analysis of ES clones by multiplex ligation-dependent probe amplification (MLPA). (A) Schematic diagram of MLPA probes for WT and null alleles of the *Cyp2j* locus and of two control probes for chromosome 8 and 11. (B) Representative MLPA data of WT, non-target (12F12), and target (4G02) ES clones. The extra 5 m, 3 m and neo amplicons were detected in DNA from non-target and target ES clones but not from WT ES clones. The relative peak areas of 5wt and 3wt were decreased by approximately half in target ES clones (4G02) but not in non-target ES clones (12F12). (C) Representative MLPA data for three middle amplicons, wtM1, wtM2, and wtM3, which were reduced by approximately half in target ES clones (4G02) as well, is shown. MLPA reactions were repeated three times for all target ES clones.

The fused BAC was electroporated into mouse ES cells and geneticin-resistant clones were screened by multiplex ligation-dependent probe amplification (MLPA) using five wild-type probes—5wt, 3wt, wtM1, wtM2 and wtM3, located within the region targeted for deletion of the *Cyp2j* gene locus, and three mutant probes—5 m, 3 m and neo—located within the engineered *Cyp2j* gene locus and recognizing vector sequences and the selectable marker (Figure 2A). Two internal control probes, HP1 and ITGB3, that detect genes located on chromosome 8 and 11, respectively, were used to normalize signal intensities from probes for *Cyp2j* wild-type and mutant genes. The desired clones showed the expected pattern, in which the wild-type signal intensity (5wt, 3wt, wtM1, wtM2 and wtM3) was decreased approximately by half, indicating disruption of one allele (Figure 2B, C). The areas of mutant signal intensities, including 5m, 3m, and neo, reflect the integrated copy numbers. Clones showing the lowest mutant signal intensity among the screened clones were considered likely to be single copy integrations (representative data shown in Figure 2B).

The selectable markers and vector sequences were removed from two ES clones, 1C04 and 4G02, using R4 integrase. A plasmid expressing the integrase under the control of the chicken actin-CMV hybrid promoter was transfected into the two ES cell lines. The action of the R4 integrase resulted in excision of the sequences located between the R4 *attB* and *attP* sites, as illustrated in Figure S2. The recombination between *attB* and *attP* sites gives rise to a chromosomal R4 *attL* site (sequences shown in Figure S2) and a circularized *attR* remnant that has no mechanism for persistence during cell division. The deletion events were initially identified by PCR using primers P13 and P14. Thirty of 43 clones for 1C04 and 29 of 37 clones for 4G02 showed the expected 561 bp PCR fragment (data not shown). Successful R4 integrase-mediated recombination was confirmed by sequencing the PCR products to detect the presence of the R4 *attL* site and by MLPA to confirm loss of the geneticin resistance allele (data not shown).

### Generation of *Cyp2j*-null mice using mouse *Cyp2j* targeted ES cells

Four clones of mouse *Cyp2j* target ES cells were microinjected into C57BL/6 blastocysts. Four chimeric mice were born from 4 clones of which one, from B6-white ES cells, showed germ line transmission: among 20 litters, 20 pups from 148 offspring (13%) were derived from ES cells. Heterozygous mice were mated to generate homozygous mutant mice (*Cyp2j^−/−^*) and wild-type littermates (*Cyp2j^+/+^*). Genotyping by MLPA showed the absence of all internal regions located in the deletion region of the homozygotes (Figure S3A). Mouse genotypes were also tested by PCR, as shown in Figure S3B.

#### Generation of human CYP2J2 (hCYP2J2) transgenic mice using a modified BAC

**Figure 3:**
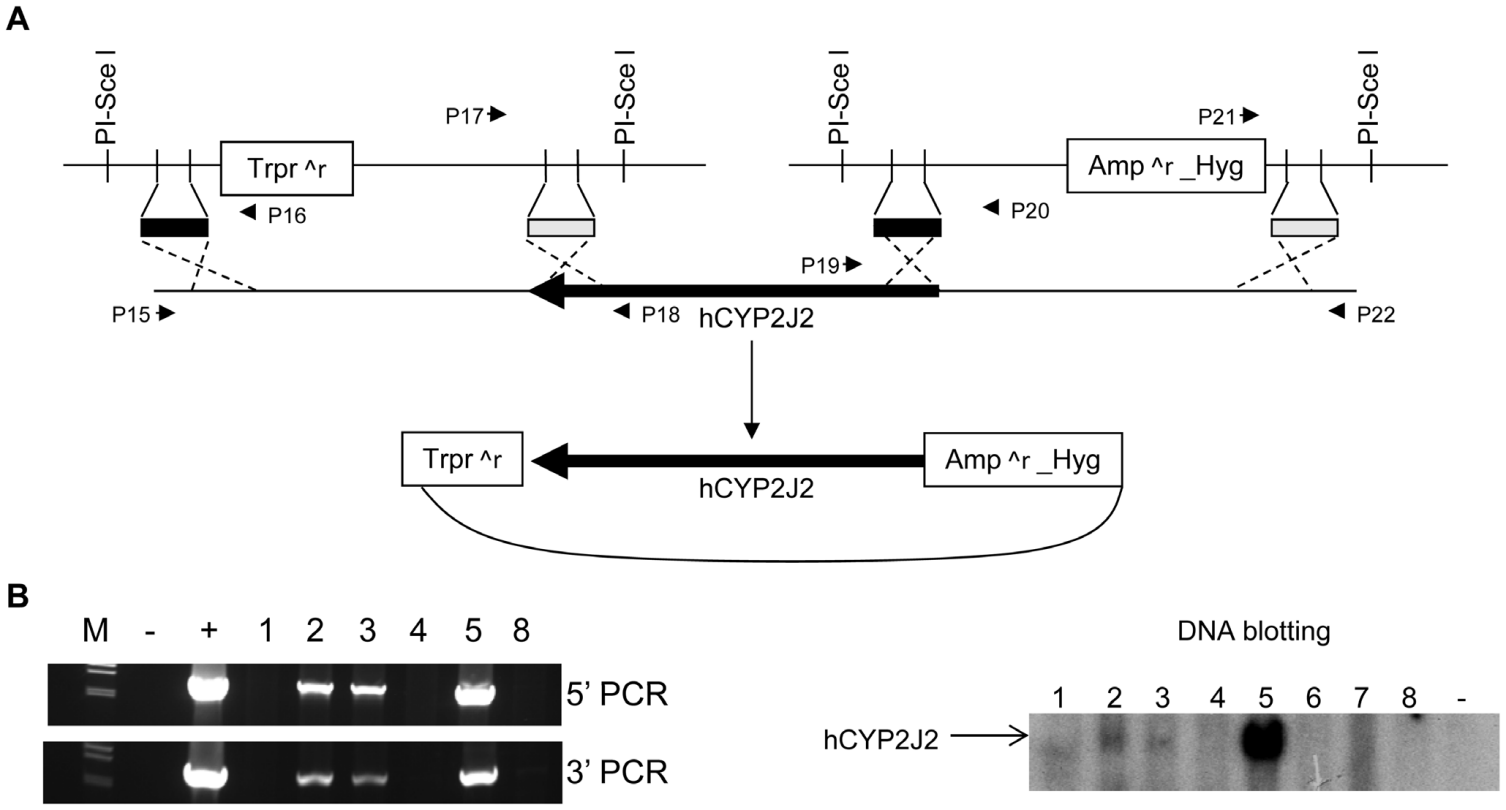
Creation of human *CYP2J2* transgenic mice. (A) Schematic diagram showing generation of the recombinant *hCYP2J2* BAC. Two targeting vectors were constructed to remove the sequences flanking *hCYP2J2* by homologous recombination in *E. coli*. Primers P15 to 22 were used to identify the recombinants in E. coli. PCR data are shown in Figure S5. Trpr∧r, trimethoprim resistance; Amp∧r, Ampicillin resistance; Hyg, Hygromycin. (B). Transgenic mice were identified by PCR and confirmed by DNA blotting. M, λ DNA marker/*Hin*d III; −, negative PCR control; +, positive PCR control to amplify BAC; 1 to 5, 8, founder mice.

A BAC clone was selected to provide the human *CYP2J2* gene. A schematic diagram shows the procedure used to modify the BAC (Figure 3A). The *E. coli* recombination events were identified by four PCR amplifications using primers P15 to P22 shown in Figure 3A (Figure S4A). The PCR products were sequenced to confirm the anticipated recombination events. The correct recombinants were digested using *Eco*RI, *Nco*I, and *Hin*dIII, separately, to confirm the identity of the sequence (Figure S4B). The recombinant BAC DNA was used to produce *hCYP2J2* transgenic mice (C57BL/6 background). Four founders were identified from 35 offspring by DNA blotting and PCR (Figure 3B). Three founders supported germ line transmission. These mice were backcrossed with *Cyp2j^−/−^* mice for more than 6 generations to derive *Cyp2j^−/−^* mice on the B6-white background carrying a transgene specifying human *CYP2J2* (*Cyp2j^−/−^-Tg*).

### *Cyp2j* gene expression in *Cyp2j^−/−^* and *Cyp2j^−/−^*-Tg mice

**Figure 4:**
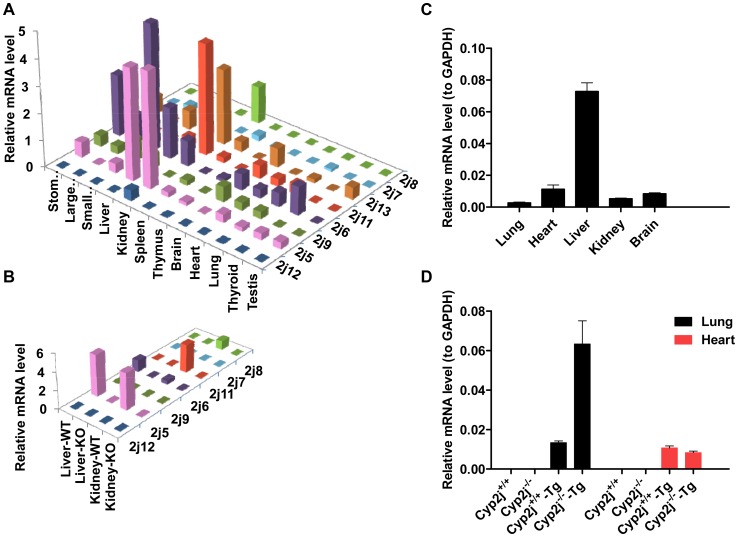
Representative data for quantitation of *Cyp2j* gene expression. (A) Mouse *Cyp2j* gene expression in different tissues was measured using RT-MLPA. (B) Mouse *Cyp2j* gene expression in liver and kidney of wild-type (WT) and null (KO) mice measured using RT-MLPA is shown. (C) *CYP2J2* gene expression in human tissues. (D) Human *CYP2J2* mRNA levels in lung and heart of *Cyp2j* ^+/+^, *Cyp2j^−/−^* , *Cyp2j^+/+^* -*Tg* and *Cyp2j^−^* ^/−^-*Tg* mice quantified by RT-PCR are shown. The measurements were performed three times using pooled mouse RNA from three individual mice.

Human CYP2J2 and the eight mouse Cyp2js share 66–83% similarity in protein sequence and 55–88% sequence identity for mRNA sequence (Figure S5). RT-MLPA [20], a technology which allows detection and quantitation of nucleic acids having single nucleotide differences, as well as measurement of the expression of multiple genes in a single tube, was used to examine the expression of the eight *Cyp2j* genes in wild-type mice. Expression of 3 internal control genes, *Tbp*, *Hprt*, and *Gapdh*, was used to normalize the data. Each *Cyp2j* gene has a distinct expression pattern, as shown in Figure 4A. Kidney, liver, and gastrointestinal tissues are the major sites of *Cyp2j* isoform gene expression. Expression of six *Cyp2j* genes (*2j5*, *2j6*, *2j8*, *2j11*, and *2j13*) is detectable in liver and kidney. *Cyp2j7* is expressed at low levels in the liver. *Cyp2j13* is highly expressed in the kidney. *Cyp2j9* shows expression in small intestine, liver, and brain. *Cyp2j6* is broadly expressed: small intestine > stomach > thyroid > liver > large intestine > kidney > brain. Only low levels of expression of *2j5*, *2j6*, *2j9*, *2j11*, and *2j13* were detected in lung. RT-MLPA was also applied to RNA prepared from liver and kidney of *Cyp2j^−/−^* mice. No transcripts from *Cyp2j* genes were detected (Figure 4B).

To evaluate potential species differences in lineage-dependent expression, *CYP2J2* gene expression was examined by quantitative reverse-transcriptase PCR (RT-PCR) using commercial pooled human cDNA preparations. The human gene is highly expressed in liver, and the abundance of transcripts in heart exceeds that in lung (Figure 4C). In contrast, in RNA prepared from *Cyp2j^−/−^-Tg* mice, CYP2J2 mRNA levels were substantially higher in lung than in heart in *Cyp2j^−/−^-Tg* mice but not in *Cyp2j^+/+^-Tg* mice (Figure 4D), an observation that was verified in mice derived from two independent founders (data not shown).

### Effects of *Cyp2j* deletion on hemodynamic parameters

To investigate whether *Cyp2j* deficiency affects systemic hemodynamic measurements, the blood pressure (BP) and heart rate (HR) were measured in conscious male and female *Cyp2j^+/+^* and *Cyp2j^−/−^* mice by tail cuff plethysmography. Systemic blood pressure and HR did not differ between genotypes (Table 1). Invasive hemodynamic measurements in anesthetized *Cyp2j^+/+^* and *Cyp2j^−/−^* mice of both sexes also did not reveal differences in HR, BP, cardiac output, systemic vascular resistance, or left ventricular systolic or diastolic function (Table 2).

**Table 1:** Comparison of systemic hemodynamic measurements in conscious *Cyp2j^+/+^* and *Cyp2j^−/−^* mice.

Genotype	HR (beats·min^−1^)	SBP (mmHg)
*Cyp2j* ^+/+^ male	580±42	114±5
*Cyp2j* ^+/+^ female	542±25	113±5
*Cyp2j* ^−/−^ male	508±34	104±3
*Cyp2j* ^−/−^ female	556±19	110±4

Data are means ± SEM. *Cyp2j^+/+^* male (n = 7), *Cyp2j^+/+^* female (n = 10), *Cyp2j^−/−^* male (n = 7), and *Cyp2j^−/−^* female (n = 9) mice. HR, heart rate; SBP, systolic blood pressure.

**Table 2:** Comparison of systemic hemodynamic measurements in anesthetized *Cyp2j^+/+^* and *Cyp2j^−/−^* mice.

Genotype	*Cyp2j^+/+^*	*Cyp2j^−/−^*
HR, beats·min^−1^	587±6	593±12
LVESP, mmHg	99±2	98±2
LVEDP, mmHg	5±0.5	6±0.2
CVP, mmHg	8±0.2	9±0.3
SVR, mmHg·ml^−1^·min^−1^	9±0.4	9±1
EF, %	58±1	57±2
CO, ml·min^−1^	9±0.5	10±1
SV, µl	16±1	17±1
dP/dt_max_, mmHg·s^−1^	9471±576	9149±474
dP/dt_min_, mmHg·s^−1^	−10009±633	−10556±515
τ, ms	6±0.2	6±0.2
SW, mmHg·µl	1323±82	1327±135
Ea, mmHg·µl^−1^	6±1	6±1

Data are means ± SEM. HR, heart rate; LVESP, left ventricular end-systolic pressure; LVEDP, left ventricular end-diastolic pressure; CVP, central venous pressure; SVR, systemic vascular resistance; EF, ejection fraction; CO, cardiac output; SV, stroke volume; dP/dt_max_, maximum rate of developed left ventricular pressure; dP/dt_min_, minimum rate of developed left ventricular pressure; τ, time constant of isovolumic relaxation; SW, stroke work; Ea, arterial elastance; (n = 6 per group).

### Contribution of *Cyp2j* to hypoxic pulmonary vasoconstriction

**Figure 5:**
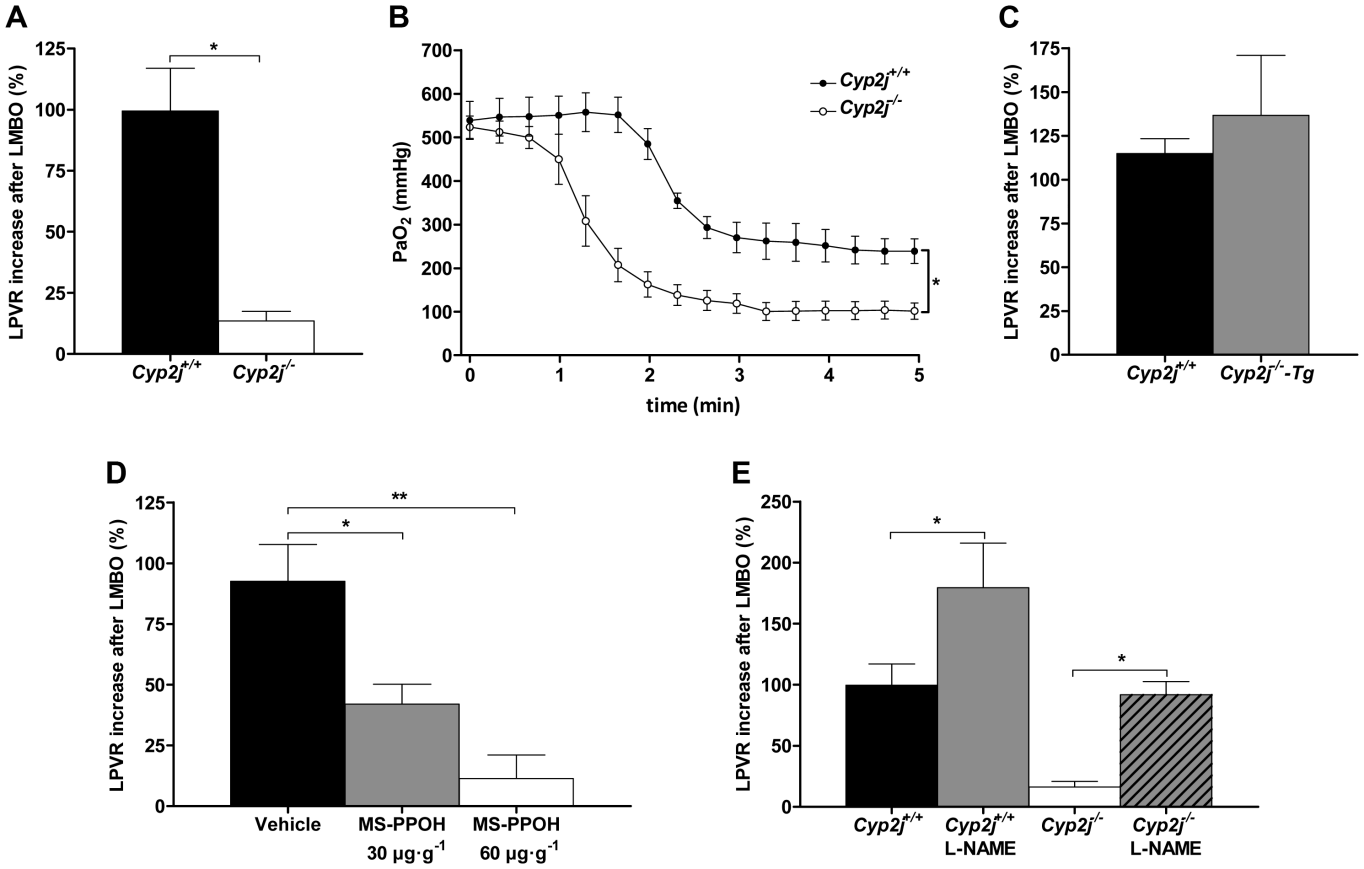
(A) Percent increase in left lung pulmonary vascular resistance (LPVR) in response to left mainstem bronchial occlusion (LMBO) in *Cyp2j^+/+^* and *Cyp2j^−/−^* mice (n = 10 per group). (B) Continuous tracings of arterial oxygen partial pressure (PaO_2_) measurements during LMBO in *Cyp2j^+/+^* (n = 4) and *Cyp2j^−/−^* (n = 3) mice; (C) Percent increase in LPVR in response to LMBO in *Cyp2j^+/+^* (n = 6) and *Cyp2j^−/−^-Tg* (n = 5) mice; (D) Percent increase in LPVR in response to LMBO in MS-PPOH or vehicle-treated *Cyp2j^+/+^* mice (n = 5 per group); (E) Percent increase in LPVR in response to LMBO in L-NAME-treated *Cyp2j^+/+^* (n = 5) and *Cyp2j^−/−^* (n = 6) and untreated *Cyp2j^+/+^* and *Cyp2j^−/−^* mice (n = 10 per group); Data are means ± SEM. *P<0.05, **P<0.005.

To assess the contribution of *Cyp2j* to HPV, we measured changes in the left pulmonary vascular resistance (LPVR) in response to left mainstem bronchial occlusion (LMBO) in *Cyp2j^+/+^* and *Cyp2j^−/−^* mice. We used dynamic measurements of pulmonary pressure and flow in the left pulmonary artery during transient inferior vena cava occlusion to estimate the LPVR [16]. Before LMBO, LPVR was similar in *Cyp2j^+/+^* (80±5 mmHg⋅ml⋅min⋅g^−1^) and *Cyp2j^−/−^* mice (88±6 mmHg⋅ml⋅min⋅g^−1^). In *Cyp2j^+/+^* mice, LMBO decreased the left pulmonary arterial blood flow (Q_LPA_) without changing the pulmonary arterial pressure (PAP), doubling the LPVR (Figure 5A, Table S3). In contrast, LMBO did not change LPVR in *Cyp2j^−/−^* mice (Figure 5A, Table S3), consistent with the absence of HPV. To estimate the impact of impaired HPV on systemic arterial oxygenation, we measured arterial blood gas tensions 5 minutes after LMBO, while the right lung was ventilated at an inspired oxygen fraction (F_I_O_2_) of 1. Arterial oxygen partial pressure (PaO_2_) was higher in *Cyp2j^+/+^* than in *Cyp2j^−/−^* mice (247±36 vs. 153±9 mmHg, respectively; *P*<0.05; Table S3). However, there was no difference in blood pH_a_, the arterial partial pressure of carbon dioxide, or the concentration of HCO_3_^−^ (data not shown). Systemic oxygenation during LMBO was further assessed using an intra-arterial PaO_2_ probe in a subset of *Cyp2j^+/+^* and *Cyp2j^−/−^* mice. No difference in PaO_2_ before LMBO was detected between *Cyp2j^+/+^* and *Cyp2j^−/−^* mice (Figure 5B). After LMBO, PaO_2_ decreased in both genotypes to its new steady state within 2 min; however, *Cyp2j^−/−^* mice had a lower PaO_2_ than did *Cyp2j^+/+^* mice during LMBO (Figure 5B). These observations confirm the presence of increased intrapulmonary shunting during LMBO in *Cyp2j^−/−^* mice and are consistent with absent HPV.

### Human *CYP2J2* restores HPV in *Cyp2j^−/−^* mice

Since *CYP2J2* is the only human gene homologous or paralogous to multiple murine *Cyp2j* genes, we investigated whether or not complementation with *CYP2J2* could restore HPV in *Cyp2j^−/−^* mice. At baseline, hemodynamic parameters did not differ between *Cyp2j^+/+^* and *Cyp2j^−/−^-Tg* mice (Table S3). LMBO increased LPVR in *Cyp2j^+/+^* and *Cyp2j^−/−^-Tg* mice to a similar extent (Figure 5C), indicating that HPV is preserved in *Cyp2j^−/−^-Tg* mice. Furthermore, during LMBO, arterial oxygen partial pressure (PaO_2_) did not differ between *Cyp2j^−/−^-Tg* and *Cyp2j^+/+^* mice (Table S3), consistent with preserved HPV. These results suggest that presence of the single human CYP2J isoform in mice, in which the entire *Cyp2j* locus is deleted, is sufficient to permit pulmonary vasoconstriction.

### Inhibition of cytochrome-P450 epoxygenase activity attenuates HPV

To exclude the possibility that the lifelong *Cyp2j* deficiency might lead to unanticipated compensatory mechanisms that could impair HPV in mice, we assessed HPV in *Cyp2j^+/+^* mice treated with the epoxygenase inhibitor, N-methylsulfonyl-6-(2-propargyloxyphenyl) hexanamide (MS-PPOH). The LMBO-induced increase in LPVR was markedly attenuated in a dose-dependent manner when mice were studied 90 minutes after treatment with MS-PPOH (Figure 5D). The PaO_2_ during LMBO was lower in MS-PPOH-treated than in vehicle-treated mice. These results further confirm that cytochrome P450 epoxygenase enzymatic activity contributes to HPV in mice.

### L-NAME restores HPV in *Cyp2j^−/−^* mice

To examine the possibility that HPV is impaired in *Cyp2j^−/−^* mice due to an alteration in the balance of vasoconstrictors and vasodilators, we studied the effects of enhancing pulmonary vascular tone by inhibiting nitric oxide (NO) production on HPV in *Cyp2j^−/−^* mice. At 30 minutes after administration of N^G^-nitro-L-arginine methylester (L-NAME, an inhibitor of NO synthases), before LMBO, hemodynamic parameters did not differ between *Cyp2j^+/+^*and *Cyp2j* ^−/−^ mice (Table S3). During LMBO, inhibition of NO synthesis with L-NAME augmented the increase in LPVR in *Cyp2j^+/+^* mice and restored the ability of LMBO to increase LPVR in *Cyp2j^−/−^* mice (Figure 5E, Table S3). These findings demonstrate that the *Cyp2j^−/−^* mice retain the mechanisms necessary for the pulmonary vascular response to hypoxia and that HPV can be restored in these mice by enhancing vasoconstriction.

### EET levels in bronchial alveolar lavage fluid and oxygenase activity in pulmonary microsomes

**Figure 6:**
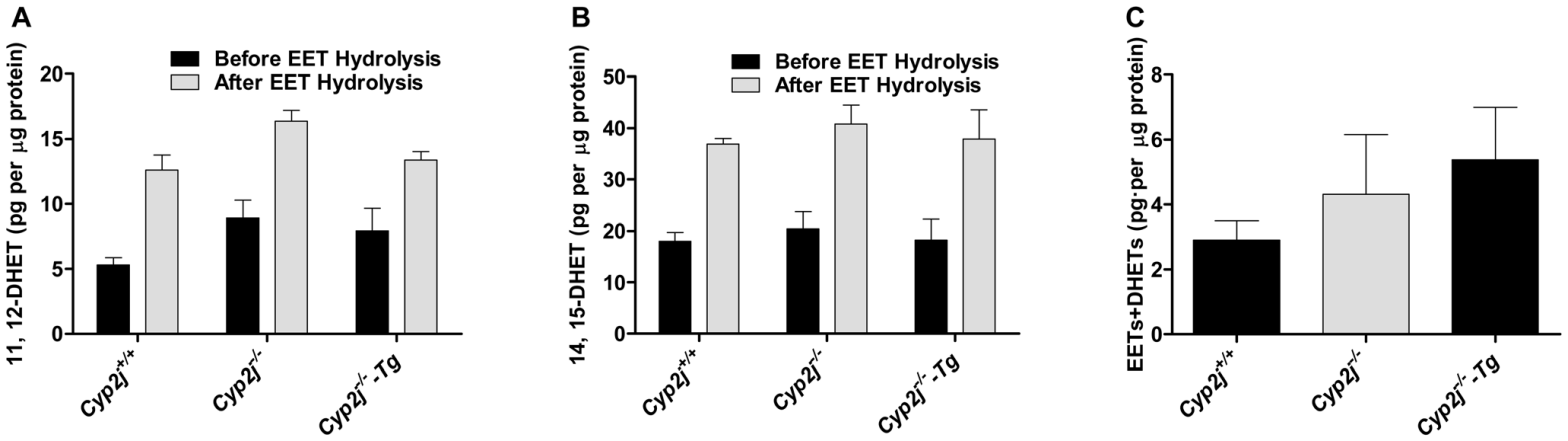
11, 12- and 14, 15-EETs measurements in BALF (A, B) and the generation of EETs and DHETs by pulmonary microsomes (C) of *Cyp2j^+/+^*, *Cyp2j^−/−^* and *Cyp2j^−/−^-Tg* mice. The black bar represents the DHET quantity before EET hydrolysis, and the grey bar represents the DHET quantity after EET hydrolysis in the samples. Triplicate measurements were performed for each mouse. Data are means ± SEM. n = 3 for each group in A, B; n = 4 for each group in C.

The plasma and urine EET levels did not differ between *Cyp2j^+/+^*and *Cyp2j^−/−^* mice (data not shown). Levels of 11, 12- and 14, 15-EET, as reflected by the difference of 11, 12- and 14, 15-DHET levels before and after hydrolysis of EETs (measured by ELISA) were evaluated in bronchoalveolar lavage fluid (BALF) from *Cyp2j^+/+^*, *Cyp2j* ^−/−^ and *Cyp2j^−/−^-Tg* mice. BALF EET levels did not differ among the genotypes (Figure 6A, B). Moreover, the generation of EETs and DHETs by pulmonary microsomes from *Cyp2j^+/+^*, *Cyp2j* ^−/−^ and *Cyp2j^−/−^-Tg* mice did not differ (Figure 6C).

### *Cyp2c44*, *2c38*, and *2c29* gene expression in lung and heart of *Cyp2j^+/+^*, *Cyp2j^−/−^* and *Cyp2j^−/−^*-Tg mice

**Figure 7:**
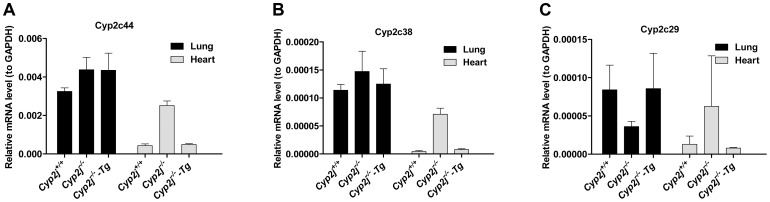
Gene expression by quantitative RT-PCR. (A, B, C) *Cyp2c44*, *Cyp2c38*, and *Cyp2c29* mRNA levels in lung and heart of *Cyp2j^+/+^*, *Cyp2j^−/−^* and *Cyp2j^−/−^ -Tg* mice. Experiments were run in triplicate. Mouse tissue RNAs were pooled from three individual mice.

In addition to members of the *Cyp2j* subfamily, members of the *Cyp2c* family of cytochrome P450 enzymes, including Cyp 2c44, 2c38, and 2c29, are able to metabolize AA to EETs in endothelial cells [21], [22]. Expression of these three Cyp2c family members in lung and heart of *Cyp2j^+/+^*, *Cyp2j* ^−/−^ and *Cyp2j^−/−^-Tg* mice was measured using quantitative RT-PCR. Pulmonary expression of the *Cyp2c* genes did not differ among genotypes, but deletion of the *Cyp2j* locus led to increased expression of the three *Cyp2c* genes in the heart (Figure 7).

## Discussion

Allelic expansion in the rodent genome is a commonly encountered phenomenon that has the potential to reduce the utility of rodent models for understanding human gene function. At a minimum, the presence of multiple functional murine paralogs confounds the extrapolation to the human context of the results of single gene ablations in mice. An alternative to single gene analysis is the inactivation of an entire locus syntenic to the human gene of interest. For the most part, the genetic tools to undertake such inactivations have been relatively underdeveloped. In this report, we demonstrate the feasibility of joining multiple bacterial artificial chromosomes using site-specific recombination to form a deletion replica that can be used to induce genomic rearrangement in mice. Previous approaches for the deletion of large DNA fragments required two targeting vectors harboring loxP sites [9]–[11]. These approaches require sequential gene targeting. The fused BAC targeting approach represents a powerful and efficient method for developing genetically-modified mice for the purpose of characterizing the function of gene clusters or studying genetic diseases associated with large chromosomal DNA deletions. Using this technology, we generated *Cyp2j*-null mice in which the 626-kb *Cyp2j* locus is deleted, as well as mice carrying a transgene specifying the human *CYP2J2* allele in context of a *Cyp2j*-null allele.

It has previously been shown that overexpression of human *CYP2J2* has cardiovascular protective effects in mice [13], [14], [23]. CYP2J synthesizes EETs in vascular endothelial cells [12], [13]. Epoxygenase-derived EETs hyperpolarize vascular smooth muscle cells in kidney, brain, and heart, resulting in vasorelaxation [24]–[27]. Previously Athiracul et al. reported that female mice deficient in the *Cyp2j5* gene on a 129/SvEv background exhibit increased systemic blood pressure [28]. We therefore expected that mice lacking the entire *Cyp2j* gene family would show systemic vascular effects. However, deletion of the *Cyp2j* locus did not affect baseline systemic hemodynamic parameters or left ventricular contractile function in either sex. The variance with previous observations might be attributable to differences in strain background or to the actions of other Cyp2j enzymes in *Cyp2j5^−/−^* mice which are not present in the *Cyp2j^−/−^* mice. Effects of *Cyp2j5* deletion on pulmonary vascular function have not been reported for *Cyp2j5^−/−^* mice.

In the pulmonary circulation, EETs enhance vasoconstrictor tone [3], [19], [29], [30], likely via activation of TRPC6 channels in vascular smooth muscle cells [30]. Epoxygenase-derived EETs are reported to contribute to the pulmonary vascular response to hypoxia [30]. Moreover, 11, 12- and 14, 15-EET levels were recently reported to be increased in isolated-perfused murine lungs following exposure to hypoxia (F_I_O_2_ 0.01) for 10 minutes [31]. Previous studies of the roles of epoxygenases in the regulation of HPV have relied on chemically-synthesized EETs, pharmacological activators or inhibitors of cytochrome P450 enzymes, or lineage-restricted overexpression of CYPs [3], [30], which cannot distinguish between the contributions of CYP2J and CYP2C. We did not detect an effect of deleting the *Cyp2j* locus on pulmonary vascular tone at baseline, but the pulmonary vasoconstrictor response to hypoxia was absent in *Cyp2j^−/−^* mice. There are several possible mechanisms by which the *Cyp2j* subfamily might regulate HPV. It is probable that *Cyp2j^−/−^* mice have reduced ability to generate pulmonary vasoconstricting EETs. Alternatively, it is possible that the deletion of *Cyp2j* subfamily shunts arachidonic acid into other pathways leading to the increased synthesis of cyclooxygenase, lipoxygenase, and ω-hydroxylase products. Some of these products, such as prostacyclin or 20-HETE are known to be pulmonary vasodilators [32], [33] and could impair HPV.

We were unable to detect differences in EET levels in the plasma, urine, and BALF of wild-type and *Cyp2j^−/−^* mice. Moreover, we did not observe differences in the generation of EETs and DHETs by microsomes extracted from the lungs of wild-type, *Cyp2j^−/−^*, or *Cyp2j^−/−^-Tg* mice. Previous studies have shown that multiple cytochrome P450 enzymes, including CYP1A, CYP2B, CYP2C, CYP2D, CYP2G, CYP2J, CYP2N, and CYP4A subfamilies, are capable of EET biosynthesis *in vitro* [34]. It is conceivable that EET production by CYP isoforms other than Cyp2j could obscure the impact of *Cyp2j* deficiency on pulmonary and systemic EET generation. In lung tissues, immunohistochemistry studies detected CYP2C proteins exclusively in the serous cells of bronchial glands, whereas CYP2J proteins were detected in a variety of cell types including pulmonary vascular smooth muscle and endothelial cells [1], [35]. We observed that *Cyp2c* genes were expressed in the lungs of wild-type and *Cyp2j* ^−/−^ mice with or without the human *CYP2J2* transgene.

In addition to actions mediated by their enzymatic activity, Cyp2j isoforms could function in signaling circuits via other mechanisms, for example, serving as scaffold proteins or mediators in signal transduction complexes that regulate HPV via mechanisms not dependent on epoxygenases or hydroxylases. The proposal that the catalytic activity of *Cyp2j* family members regulates HPV is supported by the finding of the present study that administration of an epoxygenase inhibitor, MS-PPOH, to wild-type mice impaired HPV in a dose-dependent manner.

Transgenic introduction of human *CYP2J2* into *Cyp2j*-deficient mice did not affect baseline hemodynamic parameters but restored the pulmonary vasoconstrictor response to LMBO. These results suggest that the human *CYP2J2* functions in a manner similar to one or more of the murine Cyp2j isoforms in the regulation of pulmonary vascular tone by hypoxia. It is of note that the expression of human *CYP2J2* transgene was greater in the lungs of *Cyp2j^−/−^* mice than in wild-type mice, suggesting the existence of a feedback loop designed to maintain expression of Cyp2j.

Administration of the NO synthase inhibitor, L-NAME, restored HPV in *Cyp2j*-null mice, indicating that *Cyp2j*-deficient mice retain the ability to constrict their pulmonary vasculature in response to alveolar hypoxia. This result is in agreement with observations of Ichinose et al., who reported that L-NAME restores HPV in *cPla2*-deficient mice [16]. These observations suggest that HPV is highly sensitive to the balance of vasoconstrictors and vasodilators in the lung. Enhancing vasoconstrictor tone or reducing vasodilation restores HPV in a variety of settings [36]. Taken together, these findings suggest that, although EET biosynthesis potentially increases in response to hypoxia [31] can enhance HPV, cPLA2/CYP2J signaling is not required for the pulmonary vasculature to sense and respond to regional hypoxia.

After genes duplicate, they often diverge in ways that can lead to new functions that were not exhibited by the parental gene. Mice have evolved eight *Cyp2j* genes and two pseudogenes. The results of this study have shown that the expression profile for each *Cyp2j* gene is distinct. CYP genes may also become specialized with respect to substrate specificity or product distribution. *Cyp2j5* shares the highest nucleic acid sequence similarity with the human *CYP2J2* gene, whereas *Cyp2j6* and *Cyp2j9* have the highest similarity with the sequence of the human protein (Fig. S5). It is conceivable that one or more of the mouse Cyp2j isoforms may have functions that differ from the single human CYP2J2 enzyme. However, our observations that both human and mouse CYP2J enzymes contribute to HPV suggest that the function of human CYP2J2 and one or more mouse Cyp2j isoforms has been conserved as the two genomes diverged during evolution.

In conclusion, ablation of a large gene family through fused BAC-mediated homologous recombination in ES cells has generated mice in which the 626-kb murine *Cyp2j* gene cluster was deleted. The single human ortholog/paralog *CYP2J2* was introduced transgenically to complement the deleted mouse locus. Surprisingly, genetically modulating Cyp2j activity did not affect baseline vascular function. However deletion of the *Cyp2j* gene locus resulted in a compromise of the pulmonary vasoconstrictor response to hypoxia.

## Materials and Methods

### BAC clones

Mouse BAC clones RP23-24J24 and RP23-70M4 and the human BAC clone RP11-163O24 were obtained from the Children's Hospital Oakland Research Institute.

### Sequences of oligonucleotides for PCR and probes for multiplex ligation-dependent probe amplification

Primers P1 through P22, mouse genotyping primers, and RT-PCR primer sequences are shown in Table S1. The MLPA probe sequences are shown in Table S2.

### Integrase

A codon-optimized TP901 integrase was designed by Dr. Changhong Pang and placed in an *E. coli* expression vector (pacycTP901_ermb) under the control of the *araBAD* promoter. A codon-optimized R4 integrase was inserted in the mammalian expression vector pEAK15 under the control of the chicken actin-CMV hybrid promoter.

### Animals

All the animal studies conform to the *Guide for the Care and Use of Laboratory Animals* published by the National Institutes of Health and were approved by the Subcommittee on Research Animal Care of the Massachusetts General Hospital.

### Reagents

The non-selective nitric oxide synthase (NOS) inhibitor, N^G^-nitro-L-arginine methylester (L-NAME); kanamycin; and ampicillin were purchased from Sigma-Aldrich, St. Louis, MO. The selective epoxygenase inhibitor, N-methylsulfonyl-6-(2-propargyloxyphenyl) hexanamide (MS-PPOH), was purchased from Cayman Chemical, Ann Arbor, MI. Ganciclovir and blasticidin were purchased from Novagen. Hygromycin and geneticin were obtained from Invitrogen.

### Modification of BACs flanking the *Cyp2j* locus or containing human *CYP2J2*

The mouse *Cyp2j* and human *CYP2J2* gene structures are shown in Figure 1A. Target vectors were endowed with four restriction enzyme sites to allow insertion of the recombination homology arms (Figure 1B). Arms, 200–2000 bp in length, were amplified from BAC DNA and subcloned into the desired target vector. The target vector was cut with PI-*Sce*I, and a fragment containing the homology arms and the selection cassette was recovered by gel purification and electroporated into competent cells containing the target BAC clone and a recombinase expression vector (pacycredabsce_ermb) bearing the bacteriophage λ *redα* and *β* genes under the control of the *araBAD* promoter [6]. Electrocompetent cells were prepared by growing the BAC strain bearing the recombinase expression vector in LB medium containing 0.1% arabinose. Candidate recombinant clones were identified by growth on selective medium (kanamycin or ampicillin and chloramphenicol) and screened by PCR using primers flanking the arms (P1–8) (Figure 1B). The authenticity of candidate clones was confirmed by sequencing of the resulting PCR products. DNA from a verified clone was electroporated into DH10B competent cells and individual colonies were re-streaked on different selection plates to confirm the removal of the recombinase plasmid. The resultant recombinant BACs MT5′BAC and MT3′BAC were digested with restriction enzymes chosen to distinguish the recombinant from the original BAC sequences (data not shown).

A similar process was followed to trim the sequences flanking human *CYP2J2* gene from a human BAC (Figure 3A). Homologous arms were amplified from the wild-type BAC and subcloned into two target vectors containing two different selectable markers, conferring trimethoprim and ampicillin resistance.

### Integrase-mediated fusion of two modified BACs

A plasmid expressing TP901 integrase under *araBAD* promoter control was introduced into bacteria harboring the recombinant BAC bearing the TP901 attB site. Electrocompetent cells were prepared from cells propagated in 0.1% arabinose. The recombinant BAC bearing the TP901 *attP* site and expressing ampicillin resistance was electroporated into cells that were then spread on plates containing kanamycin, ampicillin, and chloramphenicol. The fused BAC clones were screened by PCR, and PCR products were sequenced to confirm the formation of TP901 *attL* and *attR* sites. DNA was prepared from a correctly fused clone and electroporated into DH10B competent cells to remove the TP901 expression plasmid. DNA from the fused BAC was digested with diagnostic restriction enzymes to confirm the structural integrity of the fused BAC.

### Mouse ES cell culturing and targeting

ES cell lines were cultured with irradiated fibroblast feeder cells in Knock-Out Dulbecco's Modified Eagle's Medium (KO-DMEM) supplemented with 15% Fetal Bovine Serum and 1000 units Leukemia Inhibitory Factor (LIF) per mL. BAC DNA (5–20 µg) was digested with *Not*I or PI-*Sce*I in 30–50 µL volume overnight and then electroporated into 10^7^ ES cells at 0.25 kV, 960 µF with a Bio-Rad Gene Pulser. Transformants were selected for 8–10 days with 250 µg/mL geneticin (Invitrogen).

### Removal of the vector and selectable sequence by R4 integrase action in ES cells

A transfection mixture containing a plasmid capable of expressing R4 integrase and Lipofectamine 2000 (Invitrogen) was prepared according to the manufacturer's instructions and incubated for 20–30 min at room temperature. Target ES cells at 80–90% confluence were trypsinized using 0.1% trypsin in 10 mM EDTA and resuspended in fresh ES cell culture medium with low (2%) serum at 3×10^5^ cells per mL. The ES cell suspension (10 mL) was mixed with the transfection mixture and replated on a 10 cm feeder plate. After 24 h, the negative selective agent, ganciclovir (2.5 µM), was added. Transformant colonies were visible after 8–10 days of culture.

### ES clone screening by MLPA

MLPA was performed as described previously [37]. Fragment analysis was carried out on an ABI 3730XL DNA analyzer.

### Blastocyst injection and testing for germ line transmission

ES cells were injected into C57/BL6 mouse blastocysts to generate chimeric mice. Chimeras from ES cell clones derived from the B6-white ES cell line were mated with wild-type B6-white mice (B6(Cg)-*Tyr^c-2J^*/J, Jackson Laboratory, Bar Harbor, Maine, USA) to test germ line transmission identifiable by coat color difference. Heterozygous mice (*Cyp2j^+/−^*) were identified by MLPA. *Cyp2j^−/−^* and littermate-matched wild-type (*Cyp2j^+/+^*) mice were obtained by mating of pairs of *Cyp2j^+/−^* mice.

### Generation of human *CYP2J2* transgenic mice

The recombinant human *CYP2J2* BAC DNA was linearized by PI-SceI digestion. The purified DNA was microinjected into pronuclear zygotes from (C57BL/6×DBA) F1 mice and embryos were transplanted into recipients for the generation of transgenic mice. The resultant transgenic mice (*Cyp2j^−/−^-Tg*) were backcrossed with B6-white *Cyp2j^−/−^* mice for more than 6 generations prior to molecular and physiological characterization.

### RT-MLPA

The RT-MLPA probes used in this study are shown in Table S2. The RT-MLPA procedure was performed as described previously [20].

### RNA preparation and real-time quantitative PCR

Total RNA was extracted and purified using an RNeasy kit (Qiagen) from mouse tissues (6–8 week-old). The primers used are detailed in Table S1. Reverse transcription and real-time quantitative PCR (qPCR) reactions were prepared with SuperScript II Reverse Transcriptase (Invitrogen) and iQ SYBR Green Supermix (Bio-Rad) and run in triplicate on a Bio-Rad iQ5. The cDNA panels of human adult tissue were obtained from Clontech.

### Hemodynamic studies

We studied mice of both sexes with an age range of 2–5 months, weighing 20–30 g. Animals in each experimental group were matched for body weight.

### Hemodynamic measurements in conscious *Cyp2j^+/+^* and *Cyp2j^−/−^* mice

Systolic blood pressure (SBP) and heart rate (HR) were measured using a non-invasive blood pressure system (XBP 1000, Kent Scientific, Torrington, Conn) in awake *Cyp2j^+/+^* and *Cyp2j^−/−^* mice, as described previously [38].

### Hemodynamic measurements in anesthetized *Cyp2j^+/+^* and *Cyp2j^−/−^* mice

Invasive hemodynamic measurements were performed in anesthetized *Cyp2j^+/+^ and Cyp2j^−/−^* mice, as described previously [39]. Briefly, mice were anesthetized by intraperitoneal injection of ketamine (120 mg·kg^−1^), fentanyl (0.05 mg·kg^−1^), and pancuronium (2 mg·kg^−1^). After intubation, animals were mechanically ventilated inspired oxygen fraction (F_I_O_2_) 1.0, tidal volume 10 µL·g^−1^, respiratory rate 120 breaths·min^−1^, and a fluid-filled catheter was inserted into the left carotid artery for infusion of saline (0.5 µL·g^−1^·min^−1^). A second fluid-filled catheter was inserted into the right jugular vein for measurement of central venous pressure (CVP). A thoracotomy was performed, and a pressure-volume conductance catheter (Size 1F, Model PVR-1030, Millar Instruments, Inc., Houston, TX) was inserted via the apex into the left ventricle. Systemic vascular resistance (SVR) was calculated based on mean arterial pressure (MAP), CVP, and cardiac output (CO) using following formula: SVR = ([MAP-CVP]·CO^−1^). The following parameters were derived from left ventricular pressure-volume curves: LVESP, left ventricular end-systolic pressure; LVEDP, left ventricular end-diastolic pressure; EF, ejection fraction; SV, stroke volume; dP/dt_max_, maximum rate of developed left ventricular pressure; dP/dt_min_, minimum rate of developed left ventricular pressure; τ time constant of isovolumic relaxation; SW, stroke work; Ea, arterial elastance.

### Measurement of HPV and arterial blood gases in mice

To assess HPV, left lung pulmonary vascular resistance (LPVR) was estimated before and during left mainstem bronchial occlusion (LMBO) in *Cyp2j^+/+^*, *Cyp2j^−/−^*, and *Cyp2j^−/−^-Tg* mice (n = 10, 10, and 5, respectively), using methods described previously [16]. Briefly, mice were anesthetized, mechanically ventilated at F_I_O_2_ of 1.0, and then subjected to a thoracotomy. An arterial line was inserted into the right carotid artery, a custom-made catheter was placed into the main pulmonary artery, and a flow probe was positioned around the left pulmonary artery. MAP, pulmonary arterial pressure (PAP), and left pulmonary arterial blood flow (Q_LPA_) were continuously measured and recorded before and during LMBO. To estimate the LPVR, the inferior vena cava was partially occluded to transiently reduce CO until Q_LPA_ was reduced by approximately 50%. LPVR was calculated from the slope of the PAP/Q_LPA_ relationship. The increase in LPVR induced by LMBO (ΔLPVR) was obtained by calculating the change in the mean value of the PAP/Q_LPA_ slopes in each mouse. Five minutes after LMBO, arterial blood was sampled from the right carotid artery. Blood gas tension analyses were measured by using an ABL800 FLEX analyzer (Radiometer America, Inc., Westlake, USA). To further assess the impact of *Cyp2j* deficiency on systemic oxygenation during LMBO in 4 *Cyp2j^+/+^* and 3 *Cyp2j^−/−^* mice, a flexible polarographic Clark-type oxygen micro probe (0.5 mm OD; LICOX CC1.R, GMS, Kiel-Mielkendorf, Germany) was advanced into the aortic arch via the right carotid artery. Arterial oxygen partial pressure (PaO_2_) was measured in real time and recorded continuously. The PaO_2_ electrodes were calibrated before and after each experiment in air at ambient pressure according to the manufacturer's instructions.

### Effects of cytochrome-P450 epoxygenase inhibition on HPV

Wild-type (C57BL/6-W (*B6(Cg)-Tyr^c-2J^/J*)) mice received MS-PPOH (30 or 60 mg·kg^−1^ dissolved in 50 µL dimethyl sulfoxide (DMSO)) or vehicle alone (equal volume of DMSO) (n = 5 per group) via tail vein 90 minutes before measurement of ΔLPVR. The dose and timing of administration were chosen based on data published previously [3].

### Measurements of effects of NOS inhibition on HPV

ΔLPVR was measured 30 minutes after intravenous administration of L-NAME, dissolved in 50 µL vehicle (normal saline) at a dose of 100 mg·kg^−1^ in *Cyp2j^+/+^* mice (n = 5) and *Cyp2j^−/−^* mice (n = 6). The dose was chosen based on results from a previous study [40].

### Evaluation of 11, 12- and 14,15-EET in BALF by ELISA

Collection of BALF was performed as previously described [41]. To assess *in vivo* 11,12- and 14,15-EETs production, an enzyme-linked immunosorbent assay (ELISA) kit (Detroit R&D, Inc., Detroit, MI) was used to determine concentrations of the stable 11,12- and 14,15-EETs metabolites 11,12- and 14, 15-dihydroxyeicosatrienoic acid (11,12-DHET and 14, 15-DHET) in BALF of *Cyp2j^+/+^*, *Cyp2j^−/−^* and *Cyp2j^−/−^-Tg* mice (n = 3 per group). 11,12 and 14,15-DHET were quantified by ELISA according to the manufacturer's instructions and normalized by protein content in BALF samples. The difference of the quantity of DHET before and after EET hydrolysis represents EET quantity in the samples.

### Preparation of pulmonary microsomal fractions and quantification of microsomal EET and DHET synthesis

Microsomal fractions were prepared from both lungs of *Cyp2j^+/+^*, *Cyp2j^−/−^*, and *Cyp2j^−/−^-Tg* mice (n = 4 per group) as described previously [42], [43]. To determine the eicosanoids generated, samples containing microsomal fractions were incubated with 1 µg of arachidonic acid and 1mM NADPH for 1 hour at 37C in a shaking water bath. Reactions were terminated by adding 2 volume of HPLC grade methanol and stored at −80C prior to analysis to each sample. The generated eicosanoid profiles were determined by LC-MS-MS, as previously described [44].

### Statistical analysis

All data are expressed as means ± SEM. P values<0.05 were considered statistically significant. Statistical analyses were performed using Prism 5 software (GraphPad Software Inc., La Jolla, CA). For hemodynamic experiments, a two-way ANOVA with repeated measures was used to compare differences between groups. However, when the interaction P value between time and condition was significant, a one-way ANOVA with post hoc Bonferroni tests (two-tailed) for normally distributed data or a Kruskal-Wallis test (two-tailed) with a post hoc Dunn's test for data that was not normally distributed was used. Measurements within the same experimental group were compared by a paired t-test. If the normality test failed, Mann-Whitney rank sum test was applied.

## Supporting Information

Figure S1 PCR identification of recombinant BACs and enzymatic digestion of a fused BAC. (A) The recombinant MT5′BAC and MT3′BAC candidates were screened by amplifying a homology region using primers P1–8 positioned outside of the homologous recombination region (HR) (shown in Figure 1B). 1–5: Recombinant clones; − : PCR control without primers; + : PCR control to amplify HR. (B) The fused BACs (FS BAC) formed by TP901 integrase action were screened by amplifying attL1 and attR1 regions using primers P9–12 (shown in Figure 1B) and validated by sequencing of the PCR products. TP901 recognizes attB1 and attP1 sites (DNA sequences are shown below) on MT5′BAC and MT3′BAC, respectively, and mediate recombination between the consensus sequences (shown in red) on attB1 and attP1. The left side of attB1 links to the right side of attP1 to form attL1 and left side of attP1 links to the right side of attB1 to form attR1 (sequences shown below). 1–4: FS BAC clones; − : negative controls to amplify MT5′BAC and MT3′BAC. Representative sequencing data showing attL and attR are presented below the sequences. (C) FS BAC was digested by *Spe*I and *Bam*HI and fractionated by 1% agrose gel. The fragment sizes were predicted by the web map program (http://pga.mgh.harvard.edu/web_apps/web_map/start). FS BAC clones with correct restriction fragments were selected. M1: λ DNA/*Hind*III marker; M2: 1 Kb DNA ladder.
(TIF)

Figure S2 Selectable markers and other extraneous sequences were eliminated from target ES clones using R4 integrase. R4 integrase recognizes *attB2* and *attP2* sites (DNA sequences are shown below) on the engineered allele and mediate recombination between consensus sequences (boxed) of *attB2* and *attP2*. The left side of attB2 links to the right side of *attP2* causing the formation of *attL2* with deletion of the sequence between *attB2* and *attP2*. The deleted ES clones were screened by PCR using P13 and P14 primers and validated by sequence verification of the formation of the R4 *attL2* site.
(TIF)

Figure S3 Genotypes of wild-type, *Cyp2j^−/+^* and *Cyp2j^−/−^* mice were identified by MLPA (A) and PCR (B). The neo resistance element is lost in *Cyp2j^−/+^* and *Cyp2j^−/−^* mice, and all the wild-type sequences (5wt, 3wt, wtM1, wtM2, wtM3) are absent from *Cyp2j^−/−^* mice.
(TIF)

Figure S4 PCR identification of a recombinant human *CYP2J2* BAC. (A) Recombinant human *CYP2J2* BACs were screened by PCR (primers shown in Figure 2A). 1–10: Recombinant clones; − : PCR control without primers; + : PCR control to amplify HR. (B) Recombinant human *CYP2J2* BAC was digested by *Eco*RI, *Nco*I and *Hin*dIII and fractionated on a 1% agarose gel. BAC clone with correct restriction fragments was selected. M1: λ DNA/*Hind*III marker; M2: 1 Kb DNA ladder.
(TIF)

Figure S5 Protein and mRNA sequence alignment of human *CYP2J2* and eight mouse *Cyp2j* genes using ClustalW2.
(TIF)

Table S1 Primer sequences.
(DOCX)

Table S2 MLPA probes.
(DOCX)

Table S3 Hemodynamic measurements and systemic oxygenation during LMBO. Hemodynamic measurements at baseline and 5 minutes after LMBO in *Cyp2j^+/+^*, *Cyp2j^−/−^*, and *Cyp2j^−/−^* mice carrying a transgene specifying human *CYP2J2* (*Cyp2j^−/−^-Tg*). PaO_2_, arterial oxygen partial pressure during LMBO (F_I_O_2_ = 1). Data are means ± SEM. ^A^, P<0.05 vs. untreated mice of respective genotype. ^B^, P<0.05 vs. *Cyp2j^+/+^* mice in the same treatment group. ^C^, P<0.05 vs. baseline value of the same parameter in the same group. ^D^, P<0.05 vs. vehicle treated mice of respective genotype. *, P<0.05 vs. untreated *Cyp2j^+/+^* mice. ^†^, P<0.05 vs. untreated *Cyp2j^−/−^* mice. **, P<0.05 vs. vehicle-treated *Cyp2j^+/+^* mice.
(DOCX)
