# Supplementary figures and images for: Deletion of the Murine Cytochrome P450 Cyp2j Locus by Fused BAC-Mediated Recombination Identifies a Role for Cyp2j in the Pulmonary Vascular Response to Hypoxia
Source: PLoS Genet. 2013 Nov 21;9(11):e1003950. doi: 10.1371/journal.pgen.1003950 (PMC3836722; doi:10.1371/journal.pgen.1003950)

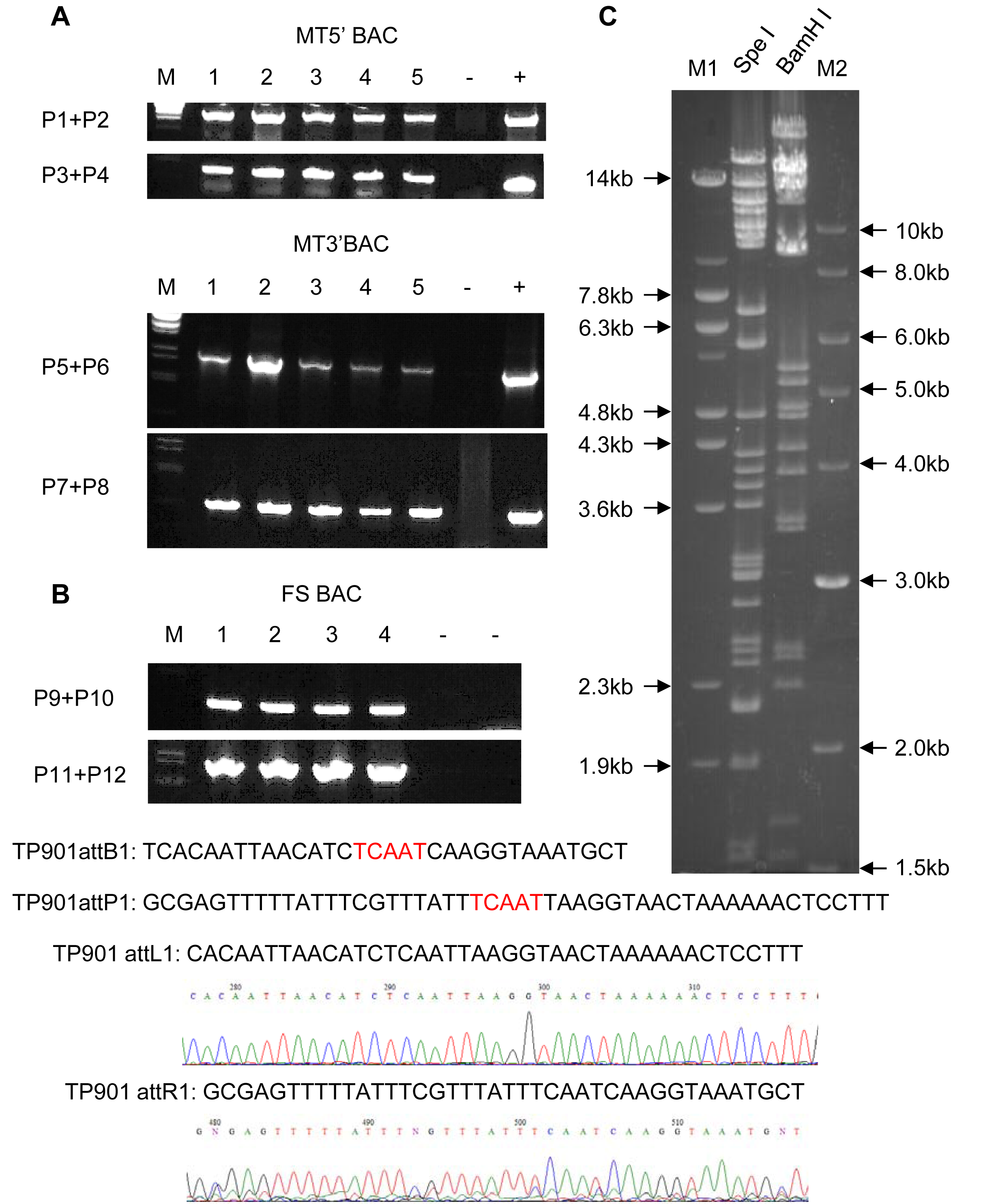

Supplement: Figure S1 — PCR identification of recombinant BACs and enzymatic digestion of a fused BAC. (A) The recombinant MT5′BAC and MT3′BAC candidates were screened by amplifying a homology region using primers P1–8 positioned outside of the homologous recombination region (HR) (shown in Figure 1B). 1–5: Recombinant clones; − : PCR control without primers; + : PCR control to amplify HR. (B) The fused BACs (FS BAC) formed by TP901 integrase action were screened by amplifying attL1 and attR1 regions using primers P9–12 (shown in Figure 1B) and validated by sequencing of the PCR products. TP901 recognizes attB1 and attP1 sites (DNA sequences are shown below) on MT5′BAC and MT3′BAC, respectively, and mediate recombination between the consensus sequences (shown in red) on attB1 and attP1. The left side of attB1 links to the right side of attP1 to form attL1 and left side of attP1 links to the right side of attB1 to form attR1 (sequences shown below). 1–4: FS BAC clones; − : negative controls to amplify MT5′BAC and MT3′BAC. Representative sequencing data showing attL and attR are presented below the sequences. (C) FS BAC was digested by SpeI and BamHI and fractionated by 1% agrose gel. The fragment sizes were predicted by the web map program (http://pga.mgh.harvard.edu/web_apps/web_map/start). FS BAC clones with correct restriction fragments were selected. M1: λ DNA/HindIII marker; M2: 1 Kb DNA ladder. (TIF) [file pgen.1003950.s001.tif]

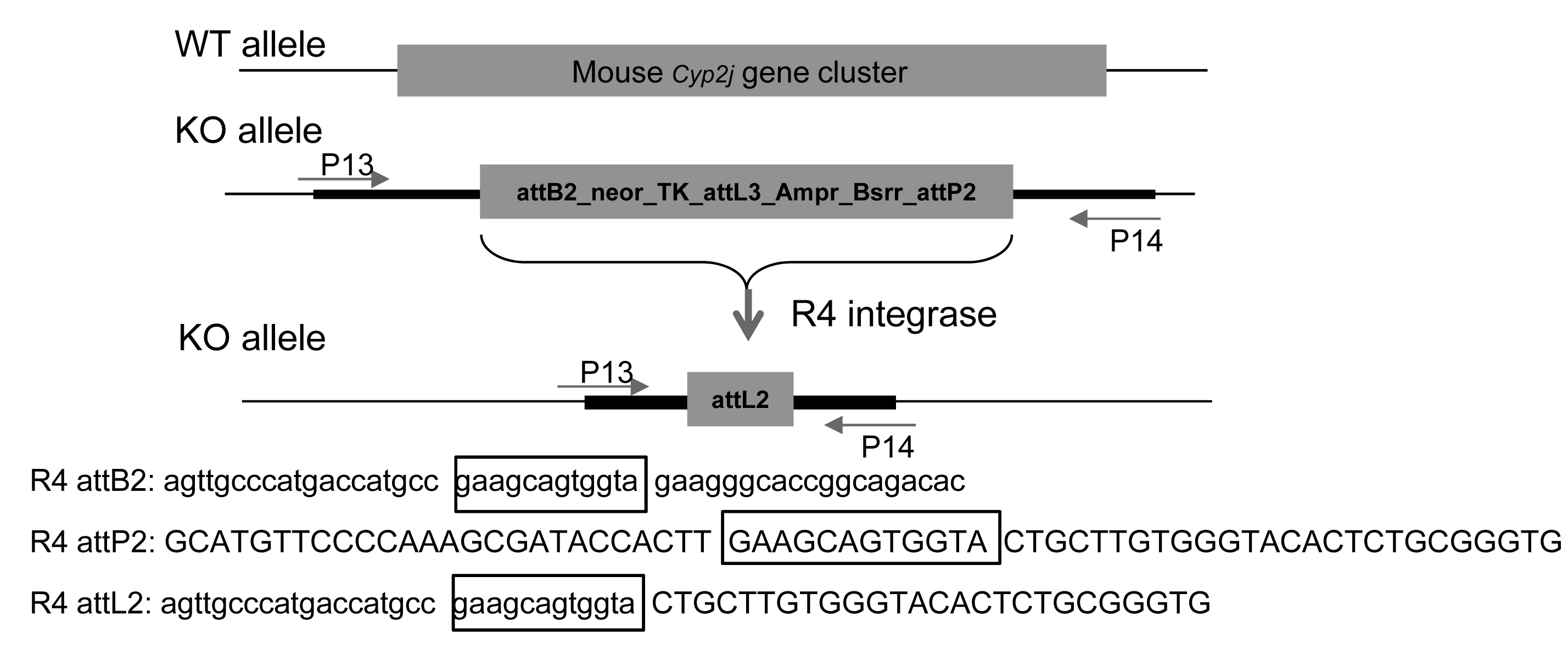

Supplement: Figure S2 — Selectable markers and other extraneous sequences were eliminated from target ES clones using R4 integrase. R4 integrase recognizes attB2 and attP2 sites (DNA sequences are shown below) on the engineered allele and mediate recombination between consensus sequences (boxed) of attB2 and attP2. The left side of attB2 links to the right side of attP2 causing the formation of attL2 with deletion of the sequence between attB2 and attP2. The deleted ES clones were screened by PCR using P13 and P14 primers and validated by sequence verification of the formation of the R4 attL2 site. (TIF) [file pgen.1003950.s002.tif]

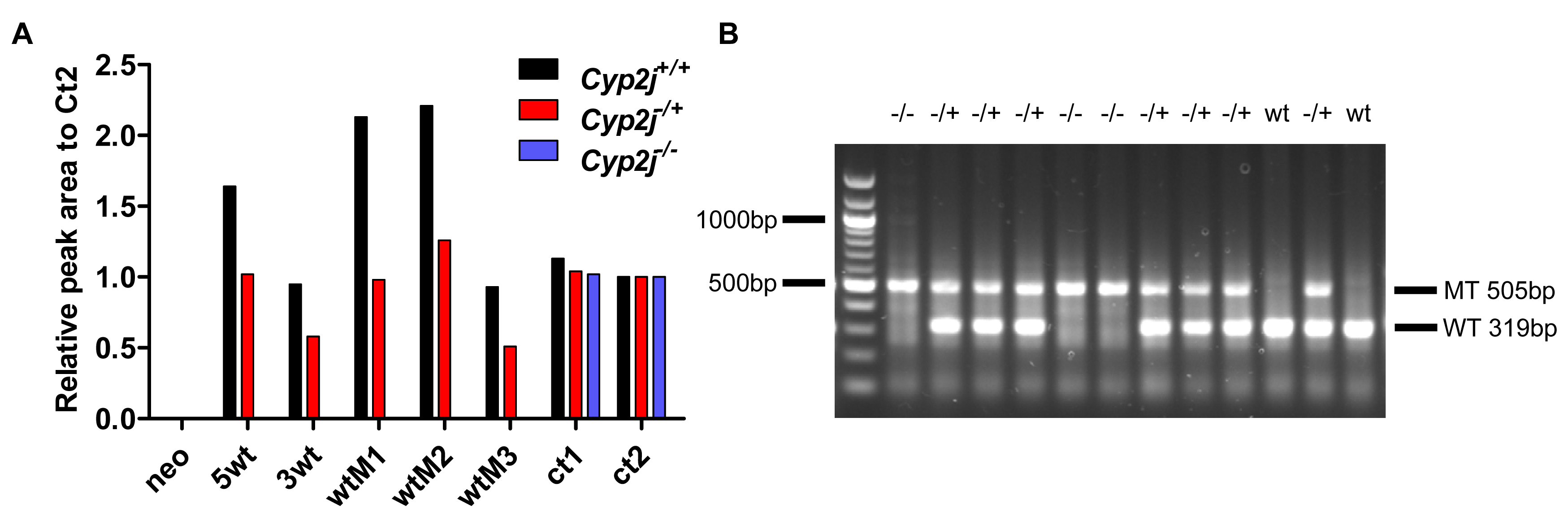

Supplement: Figure S3 — Genotypes of wild-type, Cyp2j−/+ and Cyp2j−/− mice were identified by MLPA (A) and PCR (B). The neo resistance element is lost in Cyp2j−/+ and Cyp2j−/− mice, and all the wild-type sequences (5wt, 3wt, wtM1, wtM2, wtM3) are absent from Cyp2j−/− mice. (TIF) [file pgen.1003950.s003.tif]

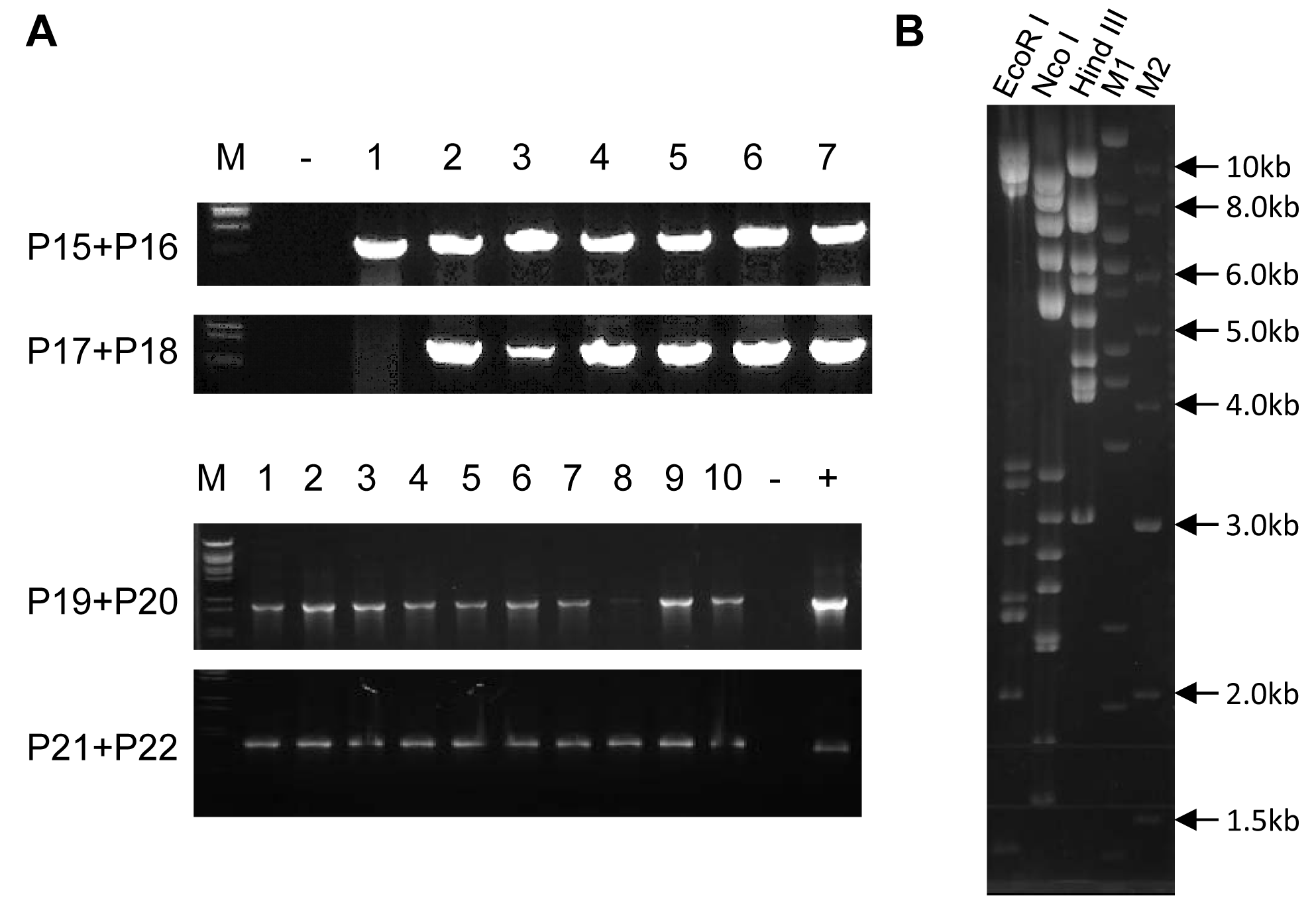

Supplement: Figure S4 — PCR identification of a recombinant human CYP2J2 BAC. (A) Recombinant human CYP2J2 BACs were screened by PCR (primers shown in Figure 2A). 1–10: Recombinant clones; − : PCR control without primers; + : PCR control to amplify HR. (B) Recombinant human CYP2J2 BAC was digested by EcoRI, NcoI and HindIII and fractionated on a 1% agarose gel. BAC clone with correct restriction fragments was selected. M1: λ DNA/HindIII marker; M2: 1 Kb DNA ladder. (TIF) [file pgen.1003950.s004.tif]

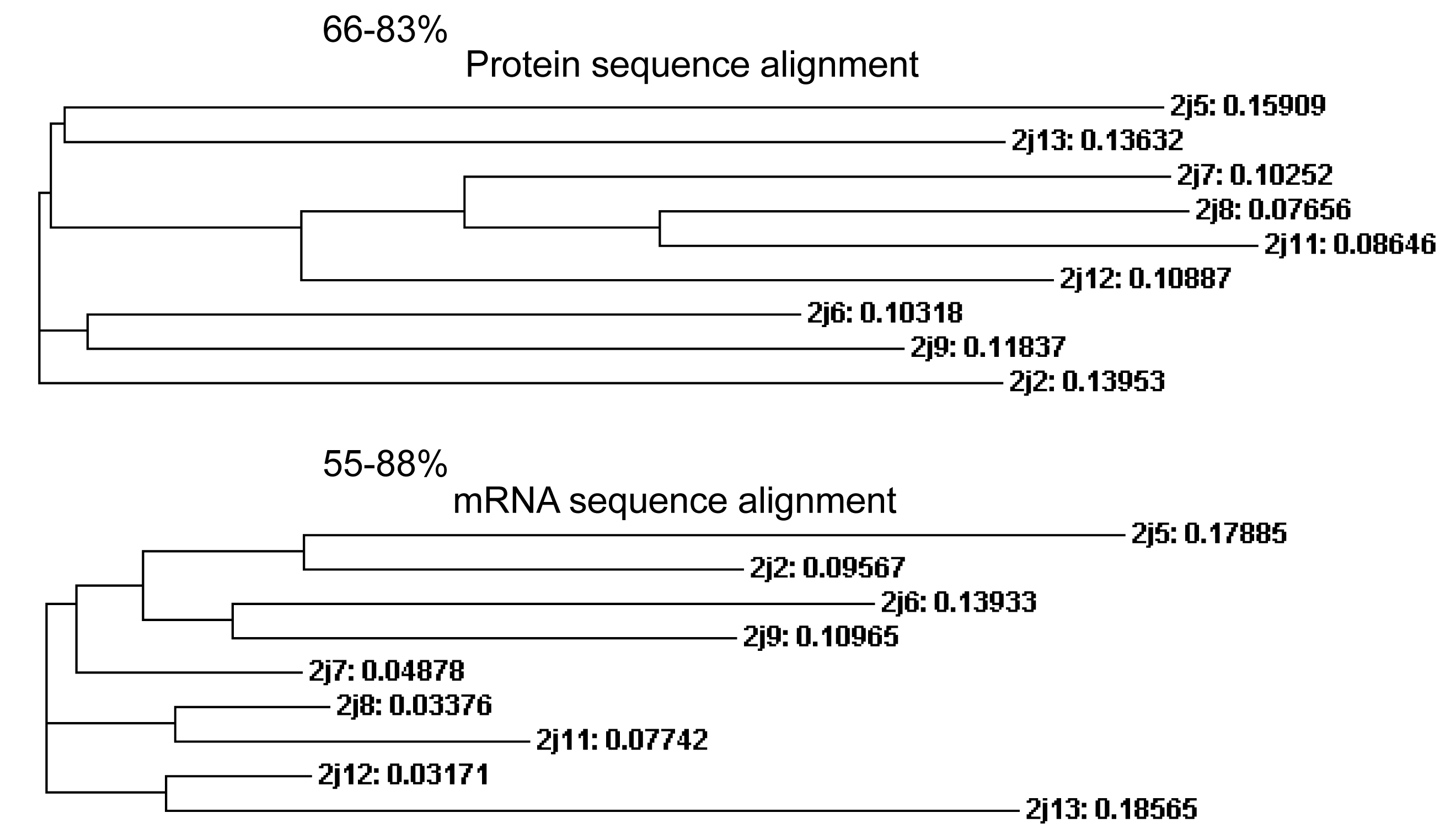

Supplement: Figure S5 — Protein and mRNA sequence alignment of human CYP2J2 and eight mouse Cyp2j genes using ClustalW2. (TIF) [file pgen.1003950.s005.tif]
