# Supplementary material for: Deletion of the Murine Cytochrome P450 Cyp2j Locus by Fused BAC-Mediated Recombination Identifies a Role for Cyp2j in the Pulmonary Vascular Response to Hypoxia
Source: PLoS Genet. 2013 Nov 21;9(11):e1003950. doi: 10.1371/journal.pgen.1003950 (PMC3836722; doi:10.1371/journal.pgen.1003950)
Supplement: Table S1 — Primer sequences. (DOCX) [file pgen.1003950.s006.docx]

| **Name** | **Sequence** | **Fragment Size (bp)** |
| --- | --- | --- |
| P1 | GAATATCATCCTGTGGAAGGCCTG | 2272 |
| P2 | GGTTACATTTGCTGTGTCATCC |  |
| P3 | CTAGTTGTGGTTTGTCCAAACTCAT | 328 |
| P4 | GCGTAACCTGGCAAAATCGGTTACG |  |
| P5 | GGATATCGTCCATTCCGACAGCATC | 1885 |
| P6 | TGTCTCATCTGTCAAGCTAGC |  |
| P7 | GTACTGCTTGTGGGTACACTC | 746 |
| P8 | CTAACAATGCCCAGTCATGGATAG |  |
| P9 | GACGCCCTGCTGCAACTTACCTC | 418 |
| P10 | GAAAATGTTGGATGCTCATACTC |  |
| P11 | CTTTCTCCGCACCCGACATAG | 2081 |
| P12 | GCGCTATATGCGTTGATGCAATTTC |  |
| P13 | CTCCTTTAGACCCACATCTATGGC | 527 |
| P14 | CAGGCAGGTTATGGTCACTGGGT |  |
| P15 | GCGCCGTTCGCTAACTCAGCATC | 1097 |
| P16 | GGCTAAATACGGAAGGATCTGAG |  |
| P17 | CTGCTTGTGGGTACACTCTGCG | 2179 |
| P18 | AGAGATGAAGAACCCCATTTGGAT |  |
| P19 | GAGGGACCCCATATTATCATTA | 2128 |
| P20 | GCTAGCGTGTCTGCCGGTGCCCTTCT |  |
| P21 | GCAAGGGGCCCAAGTTCCTATc | 1061 |
| P22 | GACCGTTCAGCTGGATATTAC |  |
| WT | CCAGAAACCAGATTAGGGCA | 319 |
|  | GACAGGGTGGAATGATGCTT |  |
| MT | GGCCCCATAGTGGACAATCT | 505 |
|  | AGGCAGGTTATGGTCACTGG |  |
| GAPDH | AGGTCGGTGTGAACGGATTTG | 123 |
|  | TGTAGACCATGTAGTTGAGGTCA |  |
| CYP2J2 | ACTGTCGCCTTTCTGCTCG | 117 |
|  | CTGCTCGAAGTCCACAAGGAA |  |
| Cyp2c44 | GGCACCGCTGTGTTCCCGTT | 180 |
|  | TCCATGCGGGCCAAACCCTC |  |
| Cyp2c38 | CCCTAAGGAATTTGGGCATGGGGA | 144 |
|  | TGACATTGCATGGAGCACAGCTCA |  |
| Cyp2c29 | CATCGACCTCCTCCCCACTAGC | 137 |
|  | GGTTGGGAAACTCCTTGCTGTCA |  |
