# Supplementary material for: Deletion of the Murine Cytochrome P450 Cyp2j Locus by Fused BAC-Mediated Recombination Identifies a Role for Cyp2j in the Pulmonary Vascular Response to Hypoxia
Source: PLoS Genet. 2013 Nov 21;9(11):e1003950. doi: 10.1371/journal.pgen.1003950 (PMC3836722; doi:10.1371/journal.pgen.1003950)
Supplement: Table S2 — MLPA probes. (DOCX) [file pgen.1003950.s007.docx]

| **Name** | **Sequence** | **Fragment Size (bp)** |
| --- | --- | --- |
| P1-Lig | GGGTTCCCTAAGGGTTGGAGGTCTGCTTCCTCCTGTGCCTGGTTAG | 103 |
| P2-NPK | TGTCTTAGTTAGGGTTTCCACTGGTTTGAAGAGACACTCCTAGATTGGATCTTGCTGGCGC |  |
| P5-NPK | GGCGCGCCCGGGAGTAGTGCCCCAACTGGGGTAACCTAGATTGGATCTTGCTGGCGCGGCGCGCCGGGAGTAGTGCCCCAACTGGGGTAAC | 107 |
| P3-Lig | GGGTTCCCTAAGGGTTGGACTTCCAAGTACAAGCCCTGCTGTGGCTTGG | 112 |
| P4-NPK | ATTACCCACTGTTGGGTGAAGCAGCCTGGTTATTTATTTCCCTAGATTGGATCTTGCTGGCGC | 127 |
| P6-Lig | GGGTTCCCTAAGGGTTGGATGAAGCAGTGGTACTGCTTGTGGGTACACTCTGCGGGTGGAATTC |  |
| P7-Lig | GGGTTCCCTAAGGGTTGGAGCTATTCGGCTATGACTGGGCACAACAGACAATCGGCT | 116 |
| P8-NPK | GCTCTGATGCCGCCGTGTTCCGGCTGTCAGCGCAGGCCTAGATTGGATCTTGCTGGCGC |  |
| P9-Lig | GGGTTCCCTAAGGGTTGGACATCTCCTTTTGCTTCAGGGGTGTACACCTTAAACGACGAGAAG | 122 |
| P10-NPK | CAATGGGTGAACACAGTCGCTGGAGAGAAACTCCCCCCTAGATTGGATCTTGCTGGCGC |  |
| P11-Lig | GGGTTCCCTAAGGGTTGGACATCATGGCTTCTGCTAAGATGACCTCTCGTCTCCCTCCAGTATGAAGAA | 137 |
| P12-NPK | TGCCTGCTTGCCCATGTTTGGCTACAAACACGTGCTGACGCTAACCCCTAGATTGGATCTTGCTGGCGC |  |
| P13-lig | GGGTTCCCTAAGGGTTGGAGAATTATGTGCTGAGCCCAGCCTGACACTCA | 103 |
| P14-NPK | GGACTGTTAAGGAGGGACCTACATTTCTACCCTAGATTGGATCTTGCTGGCGC |  |
| P15-lig | GGGTTCCCTAAGGGTTGGACCCAATAAAGCTACAGTGGCTATCTAGTAATCCCTGGAGATTC | 127 |
| P16-NPK | ATTTGTCTGCTCTTCCCTAGTCCAAGGATTACATATGCAAGGCCTAGATTGGATCTTGCTGGCGC |  |
| P17-lig | GGGTTCCCTAAGGGTTGGAGTTGATGAGAAAAGAATTAAGAAAGCTTGGTGATAAAG | 116 |
| P18-NPK | ACGAAGGAAGGAGCCAAAGCAGAGGATATTAGTGTCCCTAGATTGGATCTTGCTGGCGC |  |
| GAPDH-Lig | GGGTTCCCTAAGGGTTGGAGGCCAAGGTCATCCATGACAACTTTGGCATTGTGGAAGGGCTCATG | 135 |
| GAPDH-NPK | ACCACAGTCCATGCCATCACTGCCACCCAGAAGACTGTGGATGGCCCCCTAGATTGGATCTTGCTGGCGC |  |
| HPRT1_lig | GGGTTCCCTAAGGGTTGGAGGATTTGGAAAAAGTGTTTATTCCTCATGGACTGATTATGGACA | 125 |
| HPRT1_NPK | GGACTGAAAGACTTGCTCGAGATGTCATGAAGGAGATGGCCTAGATTGGATCTTGCTGGCGC |  |
| TBP_lig | GGGTTCCCTAAGGGTTGGACTTATGCTCAGGGCTTGGCCTCCCCA | 95 |
| TBP_NPK | CAGGGCGCCATGACTCCTGGAATTCCCCCTAGATTGGATCTTGCTGGCGC |  |
| Cyp2j12-Lig | GGGTTCCCTAAGGGTTGGACACTGCGCTGGGCTCTGCTCTACATAACTA | 100 |
| Cyp2j12-NPK | CAAACCCAGAAGTGCAAGAGAAAGTACACCTAGATTGGATCTTGCTGGCGC |  |
| Cyp2j5-Lig | GGGTTCCCTAAGGGTTGGAGAAGTGGAAGAGCTGACATGTGCTATTGAGTC | 105 |
| Cyp2j5-NPK | ACTGGGGCCTGCTCACATGAGAAGTGATAGGCCTAGATTGGATCTTGCTGGCGC |  |
| Cyp2j9-Lig | GGGTTCCCTAAGGGTTGGAGTTCAGATTCTCATTCCTCCAAGAGCCTGCTCT | 110 |
| Cyp2j9-NPK | GATATCAGCCATGCTTGCGGCAGCCGGCTCCCTAGCCTAGATTGGATCTTGCTGGCGC |  |
| Cyp2j6-Lig | GGGTTCCCTAAGGGTTGGAGAAGGGTGCCCTTGTTGTTAGCACCTGGGACTTGCCGCAGCT | 115 |
| Cyp2j6-NPK | CAGACCTTCATTCCTCAGACGAACCAGCTGCCCTAGATTGGATCTTGCTGGCGC |  |
| Cyp2j11-lig | GGGTTCCCTAAGGGTTGGACTGGAACTTAGCAGAGGGCTGTCAGTACTGTCAGGGGAC | 120 |
| Cyp2j11-NPK | TGGCACAGCTCAGACTCTCACCATGCTTGCTATTGCAACCCTAGATTGGATCTTGCTGGCGC |  |
| Cyp2j13-lig | GGGTTCCCTAAGGGTTGGAGTTGGAAGGAATTAGACTAAGGTCAGTGGACCTATATTCTGGAC | 130 |
| Cyp2j13-NPK | TGCTGAGACCTCCAAATTGGTTGTGGATAAATCACTTACATGCTCCTAGATTGGATCTTGCTGGCGC |  |
| Cyp2j7-Lig | GGGTTCCCTAAGGGTTGGAGAACTGAGACATCATCCACAGCACTGCGCTGGGCACTGCTCTATATGACA | 140 |
| Cyp2j7-NPK | CTCAACCCGGAAGTGCAAGAAAGAGTACACTCTGAGATTGATGGAGTGCCTAGATTGGATCTTGCTGGCGC |  |
| Cyp2j8-Lig | GGGTTCCCTAAGGGTTGGAGGAACAGAGACAACATCCACAGCACTGCGTTGGGCTCTGCTCTACATAACT | 145 |
| Cyp2j8-NPK | GTTAATCCAGAAGTGCAAGAAAAAGTACACTCTGAGATTGACAGAGTGATTGCCTAGATTGGATCTTGCTGGCGC |  |
