# Supplementary material for: Deletion of the Murine Cytochrome P450 Cyp2j Locus by Fused BAC-Mediated Recombination Identifies a Role for Cyp2j in the Pulmonary Vascular Response to Hypoxia
Source: PLoS Genet. 2013 Nov 21;9(11):e1003950. doi: 10.1371/journal.pgen.1003950 (PMC3836722; doi:10.1371/journal.pgen.1003950)
Supplement: Table S3 — Hemodynamic measurements and systemic oxygenation during LMBO. Hemodynamic measurements at baseline and 5 minutes after LMBO in Cyp2j+/+, Cyp2j−/−, and Cyp2j−/− mice carrying a transgene specifying human CYP2J2 (Cyp2j−/−-Tg). PaO2, arterial oxygen partial pressure during LMBO (FIO2 = 1). Data are means ± SEM. A, P<0.05 vs. untreated mice of respective genotype. B, P<0.05 vs. Cyp2j+/+ mice in the same treatment group. C, P<0.05 vs. baseline value of the same parameter in the same group. D, P<0.05 vs. vehicle treated mice of respective genotype. *, P<0.05 vs. untreated Cyp2j+/+ mice. †, P<0.05 vs. untreated Cyp2j−/− mice. **, P<0.05 vs. vehicle-treated Cyp2j+/+ mice. (DOCX) [file pgen.1003950.s008.docx]

|  |  | n | HR | | SAP | | PAP | | Q_LPA_ | | LPVR | | P_a_O_2_ |
| --- | --- | --- | --- | --- | --- | --- | --- | --- | --- | --- | --- | --- | --- |
|  |  |  | (bpm) | | (mmHg) | | (mmHg) | | (µL·min^-1^·g^-1^) | | (mmHg·min·g·mL^-1^) | | (mmHg) |
| Genotype | Treatment |  | Baseline | LMBO | Baseline | LMBO | Baseline | LMBO | Baseline | LMBO | Baseline | LMBO | LMBO |
| *Cyp2j^+/+^* |  | 10 | 567±16 | 567±13 | 94±4 | 95±5 | 20±0.4 | 21±1 | 93±3 | 45±2^C^ | 80±5 | 160±16^C^ | 247±36 |
| *Cyp2j^-/-^* |  | 10 | 553±18 | 574±18 | 106±4 | 109±4 | 20±1 | 21±1 | 90±4 | 72±5^B^ | 88±6 | 102±7^B^ | 153±9* |
| *Cyp2j^-/-^*-Tg |  | 5 | 581±12 | 581±17 | 102±5 | 100±5 | 20±0.4 | 21±2 | 106±4 | 56±7^C^ | 59±5 | 135±16^C^ | 342±50† |
| *Cyp2j^+/+^* | Vehicle | 5 | 535±19 | 520±22 | 101±5 | 99±5 | 22±1 | 23±1 | 98±4 | 53±9^C^ | 80±7 | 156±19^C^ | 307±30 |
|  | DMSO |  |  |  |  |  |  |  |  |  |  |  |  |
| *Cyp2j^+/+^* | MS-PPOH | 5 | 551±19 | 549±18 | 105±7 | 107±7 | 20±1 | 20±1 | 101±2 | 83±3^D,C^ | 81±6 | 116±13^C^ | 141±8 |
|  | (30 µg·g^-1^) |  |  |  |  |  |  |  |  |  |  |  |  |
| *Cyp2j^+/+^* | MS-PPOH | 5 | 560±10 | 516±30 | 87±3 | 85±6 | 20±1 | 21±1 | 103±3 | 98±4^D^ | 72±9 | 78±7^D^ | 135±13** |
|  | (60 µg·g^-1^) |  |  |  |  |  |  |  |  |  |  |  |  |
| *Cyp2j^+/+^* | L-NAME | 5 | 484±18 | 468±15 | 119±6^A^ | 119±4^A^ | 19±1 | 20±1 | 95±4 | 44±4^C^ | 81±5 | 226±33^A,C^ | 256±38 |
| *Cyp2j^-/-^* | L-NAME | 6 | 553±12 | 561±19 | 121±4^A^ | 121±3^A^ | 19±1 | 21±1 | 97±6 | 52±3^A,C^ | 78±4 | 150±13^C^ | 204±37 |
